# Modulating Surface Cation Concentration via Tuning the Molecular Structures of Ethylene Glycol-Functionalized PEDOT for Improved Alkaline Hydrogen Evolution Reaction

**DOI:** 10.1021/jacsau.4c00409

**Published:** 2024-07-21

**Authors:** Hsun-Hao Lin, Hsuan-I Liang, Shyh-Chyang Luo

**Affiliations:** Department of Materials Science and Engineering, National Taiwan University, Taipei 10617, Taiwan

**Keywords:** PEDOT, ethylene glycol, surface ion concentration, surface modification, hydrogen evolution reaction

## Abstract

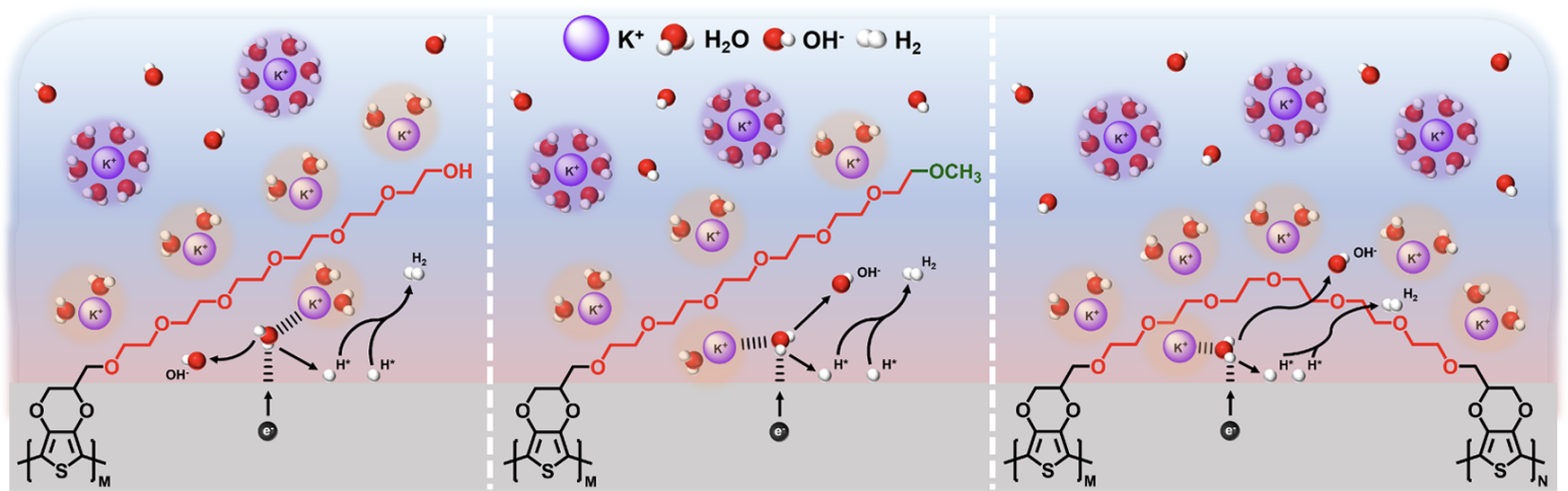

The sluggish catalytic kinetics of nonprecious metal-based electrocatalysts often hinder them from achieving efficient hydrogen evolution reactions (HERs). Poly(3,4-ethylenedioxythiophene) (PEDOT) and its derivatives have been promising materials for various electrochemical applications. Nevertheless, previous studies have demonstrated that PEDOT coatings can be detrimental to HER performance. In this study, we investigated the alkaline HER efficiency of nickel foam coated with three types of ethylene glycol (EG)-functionalized EDOT. Specifically, EDOT derivatives bearing hydroxyl (−OH) and methoxy (−OCH_3_) end groups on the EG side chain and molecules containing two EDOT units are interconnected via EG moieties. EG groups are selected due to their strong interaction with alkali metal cations. Intriguingly, improved HER performance is observed on all electrodes coated with EG-functionalized EDOTs. Electrochemical impedance spectroscopy, electrochemical quartz crystal microbalance with dissipation, and XPS analysis are employed to explore the origin of enhanced HER efficiency. The results suggest the EG moieties can induce locally concentrated ions near the electrode surface and facilitate water dissociation through noncovalent interactions. The influence of EG chain length is systematically investigated by synthesizing molecules with di-EG, tetra-EG, and hexa-EG functionalities. This study highlights the importance of molecular design in modifying electrode surface properties to promote alkaline HER.

## Introduction

Electrochemical hydrogen evolution reaction (HER) plays a crucial role in numerous sustainable energy applications. Development of the HER offers a promising pathway for generating high-purity hydrogen. Improving HER efficiency is essential for unlocking the potential of hydrogen as a clean energy source. Therefore, the design of active and cost-effective electrocatalysts has been extensively investigated. According to Sabatier’s principle for heterogeneous catalysts, the interaction between the hydrogen intermediate (H*) and the catalyst surface should be neither too strong nor too weak to facilitate the formation of M–H intermediate and the release of H_2_.^1,2^ Hence, platinum-based materials are widely considered as excellent HER electrocatalysts due to their near ideal H* adsorption Gibbs free energy.^1,3,4^ Nevertheless, the scarcity and high cost of noble metals like Pt limit their applications in scalable hydrogen production. In this case, tremendous efforts have been exerted to develop nonprecious metal-based HER electrocatalysts.^5−8^

The concentration and composition of ions in the electrical double layer (EDL) are critical factors in electrochemical processes.^9−11^ Recently, alkali metal cations in the EDL have been demonstrated to have a significant impact on the reaction kinetics of alkaline water electrolysis.^12,13^ Gao et al. reported a nanocone-assembled Ru_3_Ni catalyst that enabled an enhanced local electric field to increase the interfacial K^+^ concentration and promote the water dissociation process.^14^ Shah et al. demonstrated that small alkali cations can favor a high OH_ad_ coverage on the Pt surface in the HER potential window. The OH_ad_ in turn promoted water dissociation and Volmer-step kinetics on the Pt surface in alkaline media, leading to improved HER activity.^15^ Huang et al. proposed that the local solvation environment at the electrified interface is cation dependent. The different sizes of alkali cations can result in different interfacial hydrogen-bonding networks that can further alter the solvent reorganization energy.^16^ According to previous studies, the size and the concentration of alkali metal cations at the electrode surface can have a significant influence on the efficiency of alkaline HER.

Conducting polymers have been widely applied in HER electrocatalysts.^17−19^ Previous studies have demonstrated the applications of conducting polymers on electrode surface modification.^20−22^ Poly(3,4-ethylenedioxythiophene) (PEDOT) and its derivatives exhibit excellent electronic properties and stability and have been promising materials for a wide variety of electrochemical applications.^23−26^ The capability to adjust surface and electrochemical properties by introducing various functional groups in the EDOT molecule manifests its advantage in electrode modifications.^27,28^ In addition, the electropolymerization technique commonly employed for fabricating PEDOT films enables the rapid and large-area modification of electrodes.^29,30^

For the applications of PEDOT in HER, Winther-Jensen et al. proposed that the composite material of PEDOT and PEG can act as an efficient HER electrocatalyst.^31^ However, Gu et al. argued that the catalytic ability of the PEDOT composite originated from the underlying conductive substrate. The presence of PEDOT films can instead block the reaction active sites and result in reduced HER performance.^32^ Our previous study also suggested that when hydroxymethyl or sulfonate-functionalized EDOTs were electropolymerized on nickel foam (NF) electrodes, the HER efficiencies were reduced.^33^

**Scheme 1:**
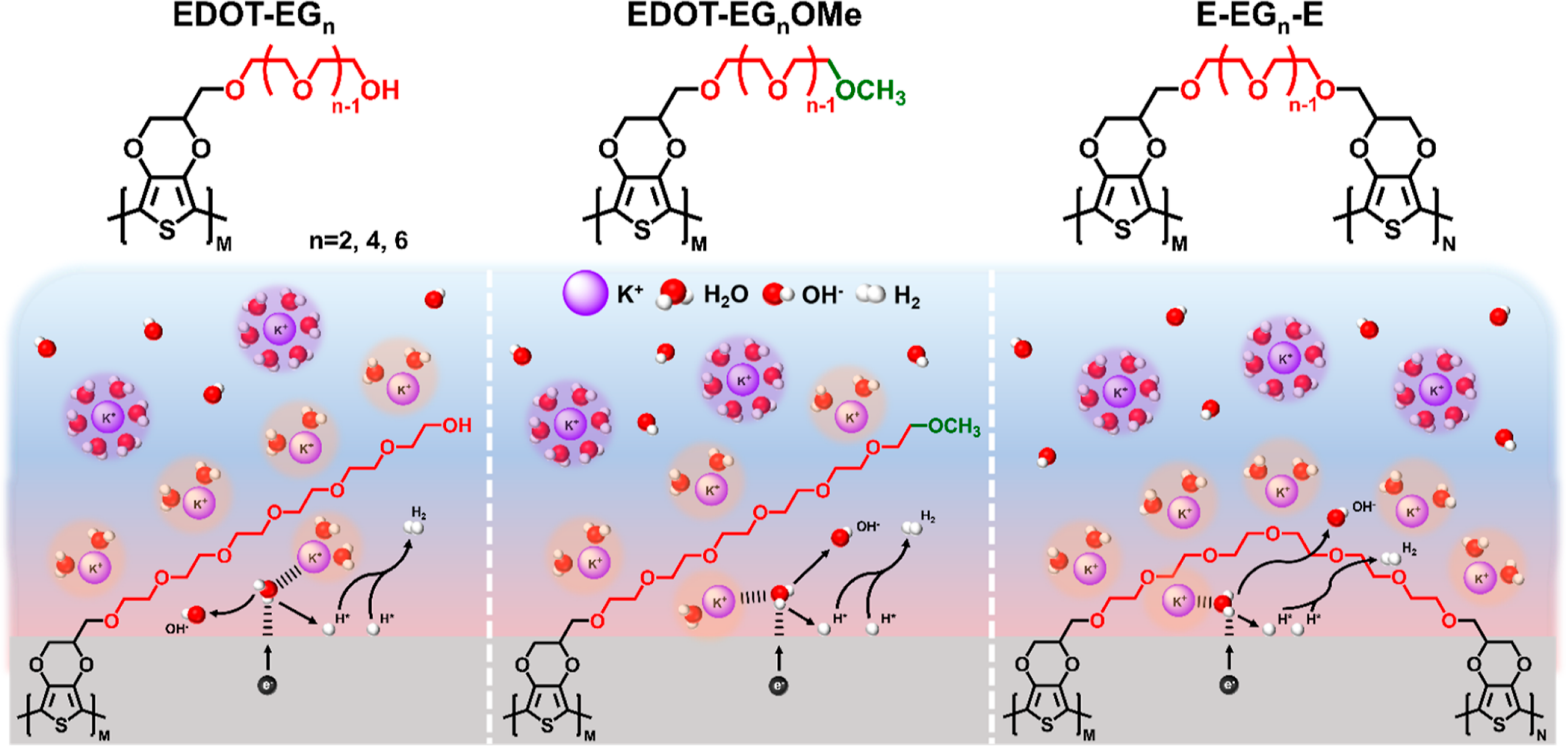
Chemical Structures of EG-Functionalized EDOTs and the Illustration of Enhanced Surface K^+^ Concentration during HER

In this study, we aimed to investigate how the molecular structures and functionalities of EDOT-based materials influence the alkaline HER performance. Specifically, three types of EDOT monomers were synthesized, and their chemical structures are shown in Scheme 1. EDOTs with hydroxyl (−OH) end group on the ethylene glycol (EG) side chain were denoted as EDOT-EG_*n*_, where *n* = 2, 4, and 6 represented the number of EG moieties on the molecules. Similarly, EDOTs with a methoxy (−OCH_3_) end group on the EG side chain were denoted as EDOT-EG_*n*_OMe. Finally, EDOT molecules containing two EDOT units bridged by an EG chain were denoted as E-EG_*n*_-E. The EG functional groups were selected in this study since EG moieties were shown to have a strong affinity toward metal cations.^34,35^ In addition, previous studies have demonstrated that the hydrophilic EG moieties can facilitate water permeation in the polymer films, leading to enhanced HER performance.^36,37^ Comparison between EDOT-EG_*n*_ and EDOT-EG_*n*_OMe were made to investigate the influence of end-group hydrophilicity, while the interconnected molecular structure of E-EG_*n*_-E was designed to introduce chemical cross-linking in the polymer film, which contributed to the film stability. The synthesized molecules were electropolymerized on NF and used as HER electrocatalysts. Surprisingly, the HER performance was enhanced after introducing EG-functionalized PEDOT films. The reaction overpotential at 50 mA cm^–2^ (η_50_) was decreased from 277.3 mV (blank NF) to 239.2 mV (EDOT-EG_2_). By replacing the Pt counter electrode with a glassy carbon (GC) electrode, we demonstrated that the superior HER performance was not due to the dissolving Pt contaminants during the reaction. In addition, linear sweep voltammetry (LSV) of NF coated with hydrophilic poly(EDOT-S) and poly(EDOT-PC) revealed that hydrophilicity was not the dominating factor for improved HER efficiency. The combination of electrochemical impedance spectroscopy (EIS), electrochemical quartz crystal microbalance with dissipation (EQCM-D), and X-ray photoelectron spectroscopy (XPS) analysis suggested that an increased cation concentration was established near the electrode surface under applied negative potential, as illustrated in Scheme 1. The elevated concentrations of cations in the local environment can enhance the polarization of the H–OH bond of the interfacial water through noncovalent interactions, leading to accelerated dissociation of water molecules, as evident from the Tafel slope and the charge-transfer resistance (*R*_ct_) from EIS measurements. The stabilities of the electrodes were also evaluated using chronopotentiometry. Superior electrode stability was observed on E-EG_6_-E due to the chemical cross-linking ability of the interconnected structure.

## Results and Discussion

### Characterization

**Figure 1:**
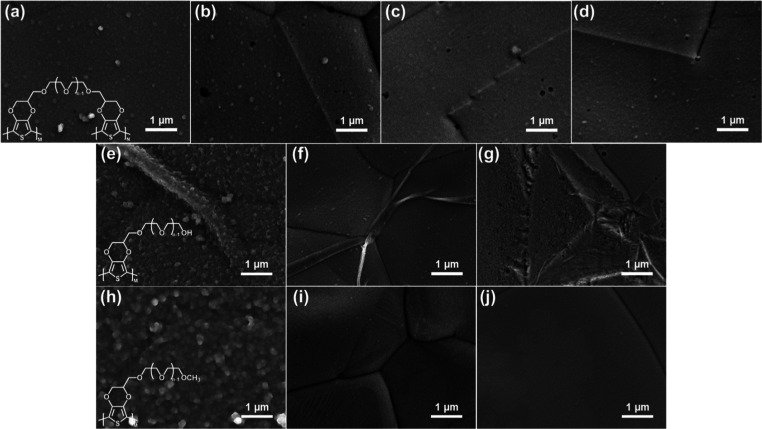
SEM images of (a) E-EG_2_-E, (b) E-EG_4_-E, (c) E-EG_6_-E, (d) E-EG_8_-E, (e) EDOT-EG_2_, (f) EDOT-EG_4_, (g) EDOT-EG_6_, (h) EDOT-EG_2_OMe, (i) EDOT-EG_4_OMe, and (j) EDOT-EG_6_OMe.

SEM images of blank NF and the electrode coated with poly(EDOT–OH) are shown in Figure S7a,b. A roughened electrode surface can be observed after the electropolymerization of EDOT–OH. The SEM images of EG-functionalized EDOTs are shown in Figure 1. Nanodot structures can be clearly seen on E-EG_2_-E, E-EG_4_-E, E-EG_6_-E, and E-EG_8_-E. The increase of EG chain length had little impact on the surface morphologies of E-EG_*n*_-E. In comparison, the surface morphologies of EDOT-EG_*n*_ and EDOT-EG_*n*_OMe demonstrated strong dependence on EG chain length. A tubular structure can be clearly seen on EDOT-EG_2_, while the structure became ambiguous as the EG chain length increased. Similarly, a nanodot structure can be observed on EDOT-EG_2_OMe, while the surface morphologies of EDOT-EG_4_OMe and EDOT-EG_6_OMe resembled the underlying poly(EDOT–OH). The strong dependence of surface morphologies on EG chain length can be ascribed to the poor film-forming ability of EDOT-EG_*n*_ and EDOT-EG_*n*_OMe with a longer EG chain. Previous publications have shown that the film-forming ability of EDOT-EG_*n*_ and EDOT-EG_*n*_OMe decreased with the increasing number of EG moieties.^38^ This can be attributed to the steric hindrance from the large side group, making it harder for the monomers to be effectively polymerized at the electrode surface.^39^ The electropolymerization of EDOT-EG_*n*_ and EDOT-EG_*n*_OMe with a longer EG chain yielded soluble products that were mostly washed away during the rinsing process. Therefore, higher potentials were applied in the electropolymerization process for EDOT-EG_*n*_ and EDOT-EG_*n*_OMe (1.4 V) compared to those for E-EG_*n*_-E (1.1 V). On the contrary, the chemical cross-linking that originated from the interconnected E-EG_*n*_-E structure can enhance the film-forming ability of the materials, leading to similar surface morphologies with varying EG chain lengths.

The high-resolution SEM images are provided in Figure S8, and the energy dispersive X-ray spectroscopy (EDS) mapping is shown in Figure S9. The S Kα1 signal can be observed on all electrodes coated with EG-functionalized EDOTs. In addition, Raman spectroscopy was conducted on EG-functionalized EDOTs (Figure S10). The characteristic peaks at 1432 and 1506 cm^–1^ were assigned to the C_α_=C_β_ symmetrical and C_α_=C_β_ asymmetrical stretching, respectively.^40,41^ The results from the experiments described above manifested the successful electropolymerization of EG-functionalized EDOTs on NF.

### Electrochemical Measurements

**Figure 2:**
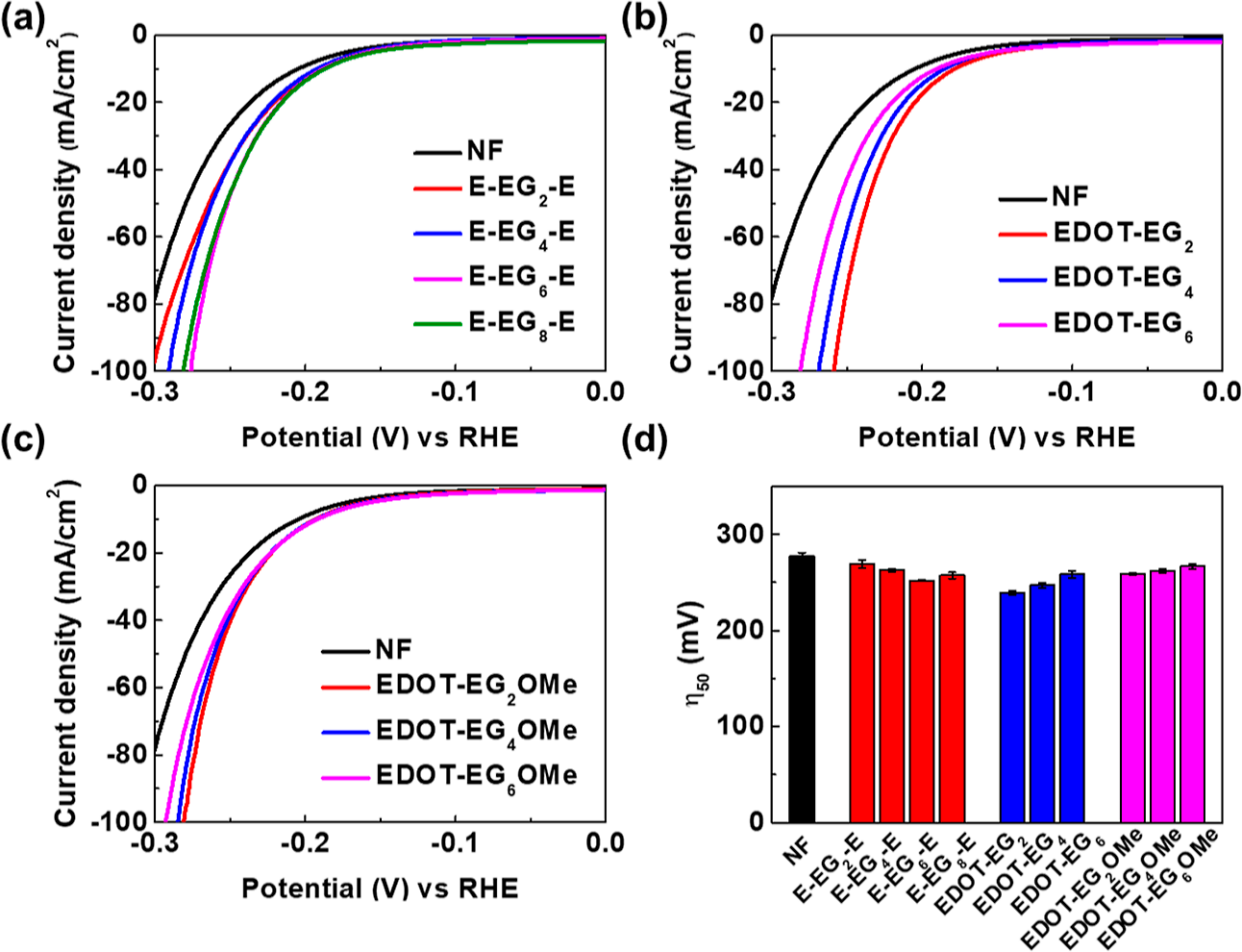
LSV results of electrodes coated with (a) E-EG_*n*_-E, (b) EDOT-EG_*n*_, and (c) EDOT-EG_*n*_OMe. (d) η_50_ values of the electrodes.

LSV experiments were carried out in 1 M KOH. The LSV curves of EG-functionalized EDOTs are shown in Figure 2a–c. All LSV measurements were repeated 3 times to ensure reproducibility. Repeated LSV curves are provided in Figure S11. The reaction overpotentials at 50 mA cm^–2^ (η_50_) are summarized in Figure 2d. The highest η_50_ value was observed on the blank NF (277.3 mV). After coating with EG-functionalized EDOTs, the reaction overpotentials were all decreased, indicating that the presence of conducting polymer films can improve HER efficiency. For E-EG_*n*_-E, the reaction overpotentials decreased with increasing EG chain length. η_50_ values were 269.3 mV, 262.6 mV, and 252.0 mV for E-EG_2_-E, E-EG_4_-E, and E-EG_6_-E, respectively. Since longer EG chains led to better HER efficiencies for E-EG_*n*_-E, a molecule with eight EG moieties (E-EG_8_-E) was synthesized to explore the optimized HER performance. The LSV curve of E-EG_8_-E is shown in Figure 2a. A slightly higher overpotential (257.2 mV) was observed compared to that of E-EG_6_-E. For EDOT-EG_*n*_ and EDOT-EG_*n*_OMe, completely opposite trends in reaction overpotentials were observed. η_50_ values were 239.2, 246.9, 258.4, 258.5, 262.2, and 266.6 mV for EDOT-EG_2_, EDOT-EG_4_, EDOT-EG_6_, EDOT-EG_2_OMe, EDOT-EG_4_OMe, and EDOT-EG_6_OMe, respectively. The reaction overpotentials increased with an increasing EG chain length. This can be attributed to the low film-forming ability of EDOT-EG_*n*_ and EDOT-EG_*n*_OMe, as evident from the SEM images. The optical images of the monomer-containing solutions before and after the electropolymerization process are shown in Figure S12. Obvious color changes can be seen after the electropolymerization process due to the formation of soluble products. As the number of EG moieties increased, the polymers became more soluble and were dissolved in the monomer solution or washed away in the rinsing process, leading to decrease in the amount of conducting polymers deposited on NF substrates. Therefore, improvement in the HER efficiency was reduced.

To elucidate the significance of film thickness in HER efficiency, E-EG_*n*_-E were electropolymerized with different cycles of the potential scan, and the LSV curves are shown in Figure S13. The HER efficiencies were improved when the potential scan was increased from 1 to 3 cycles, while decreased HER performance was observed when the potential scan was further increased to 5 cycles. Previous studies have shown that the thickness of polymer coatings can have a significant impact on HER efficiency.^32,42^ Thick polymer films can block contact between the reaction active sites and the electrolytes, resulting in poorer HER efficiency. On the contrary, if the thickness of the polymer layer was insufficient, the improvement in HER efficiency may be subtle. As a result, we can observe in Figure S13 that an optimized cycle of the potential scan during the electropolymerization process was necessary for achieving optimum HER efficiency. Therefore, 3 cycles of the potential scan were applied for all EG-functionalized EDOTs in this study.

To calculate the electrochemical active surface area (ECSA) of the electrodes, the double layer capacitance (*C*_dl_) was determined by conducting CV at different scan rates in the nonfaradaic region (Figure S14). The ECSAs of the electrodes were calculated from the measured *C*_dl_ values (Table S1).^43,44^ The ECSA values were increased after the electropolymerization of EG-functionalized EDOTs due to the enhanced roughness of PEDOT films compared to bare NF. Decreasing ECSA values can be observed on molecules with longer EG chains due to the lower amount of EDOTs electropolymerized on NF.

### Hydrophilicity of EG-Functionalized EDOTs

**Figure 3:**
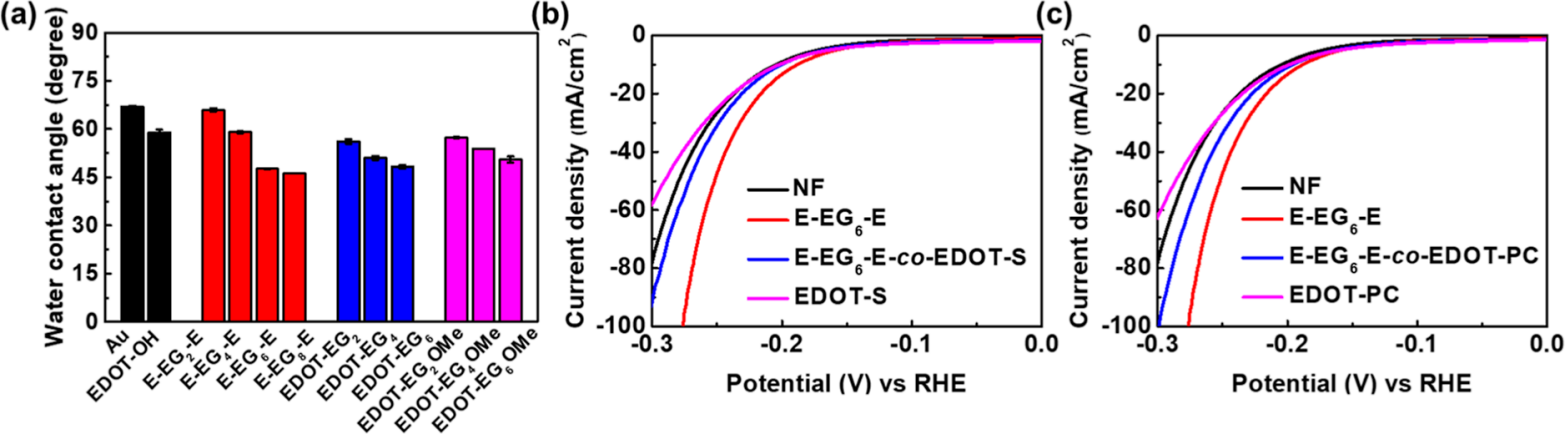
(a) Water contact angles of EG-functionalized EDOTs on Au substrate. (b) LSV results of E-EG_6_-E/EDOT-S copolymer. (c) LSV results of E-EG_6_-E/EDOT-PC copolymer.

In order to explore the underlying mechanism of the improved HER efficiency after coating NF with EG-functionalized EDOTs, our first guess is that the polymer coatings can increase the hydrophilicity and bubble-releasing ability of the electrodes, leading to reduced reaction overpotentials.^37,45,46^ Hence, we conducted water contact angle measurements for the EG-functionalized EDOTs. The contact angle of the blank NF was 121.2° (Figure S15a). After coating with poly(EDOT–OH), the electrode surface became too hydrophilic such that the water droplet fell into the pores of NF (Figure S15b). The contact angles on NF substrates cannot be measured directly. Therefore, EG-functionalized EDOTs were electropolymerized on Au substrates for contact angle measurements. The results are summarized in Figure 3a, and the optical images of water droplets are shown in Figure S16.

The contact angle of the Au substrate was 67.0°. After coating with conducting polymer films, all contact angles decreased. The polymers with longer EG chains demonstrated better hydrophilicity. The water contact angles were 66.0° for E-EG_2_-E, 59.0° for E-EG_4_-E, 47.6° for E-EG_6_-E, 46.2° for E-EG_8_-E, 56.1° for EDOT-EG_2_, 51.0° for EDOT-EG_4_, 48.3° for EDOT-EG_6_, 57.4° for EDOT-EG_2_OMe, 53.8° for EDOT-EG_4_OMe, and 50.5° for EDOT-EG_6_OMe. Comparing the water contact angles of polymers with identical numbers of EG moieties but different molecular structures, EDOT-EG_*n*_ demonstrated the lowest water contact angles. The contact angles of EDOT-EG_*n*_OMe were slightly higher, and the contact angles of E-EG_*n*_-E were the highest among the three types of polymers. This was due to the more hydrophobic methoxy and EDOT end groups in the molecular structures. Although the EG-functionalized EDOTs demonstrated better hydrophilicity than blank NF, the results of contact angle measurements were not completely consistent with the LSV experiments. In addition, previous publications have shown that EDOT coatings can be detrimental to HER efficiency regardless of the hydrophilicity of the materials.^32,33^

To gain further insight into the relation between electrode hydrophilicity and HER performance, polymers with superior hydrophilicity (EDOT-S and EDOT-PC) were employed. The water contact angles of EDOT-S and EDOT-PC on Au substrate were 22.2 and 16.4°, respectively (Figure S17). The LSV results of NF coated with poly(EDOT-S), poly(EDOT-PC), and their copolymers with E-EG_6_-E are shown in Figure 3b,c. The HER efficiencies were decreased after introducing EDOT-S and EDOT-PC, which was consistent with the results in previous publications that EDOT coatings can be harmful to HER efficiency. The deviation between the results of LSV and water contact angle measurements, together with the reduced HER efficiency after introducing hydrophilic EDOT-S and EDOT-PC, suggested that the hydrophilicity was not the main reason for the improved HER performance of EG-functionalized EDOTs.

### Insights into How EG-Functionalized EDOTs Facilitate HER

Previous studies have shown that the EG ligand environment can modulate the surface ion concentration and improve the catalytic kinetics of HER.^47^ Also, it was shown that the EG moieties in the molecular structure possess excellent adsorption capacity toward metal cations.^35^ Recent publications have demonstrated that the increase in surface cation concentration could significantly enhance HER activity by favoring the rate-determining water dissociation process (Volmer step).^14,48^ Therefore, we first tried to investigate the importance of EG moieties on the HER performance. Monomers bearing similar molecular structures with E-EG_6_-E, EDOT-EG_6_, and EDOT-EG_6_OMe but without EG functional groups (E-C_12_-E and EDOT-C_12_) were synthesized and electropolymerized on NF. The water contact angles were 81.2 and 102.5° for E-C_12_-E and EDOT-C_12_ on Au substrates, respectively (Figure S18). The LSV curves of E-C_12_-E and EDOT-C_12_ are shown in Figure S19. Obviously, the HER efficiencies were reduced after coating NF with these two polymers, indicating the importance of EG functional groups in promoting the alkaline HER.

Previous publications have demonstrated that the noble metal impurities dissolved from the counter electrodes may have a significant impact on HER efficiency.^32,49^ In this case, a GC counter electrode was used to replace Pt for the LSV measurements (Figure S20). Evidently, the reaction overpotential of E-EG_6_-E was still much lower than that of blank NF, suggesting that the improvement of HER efficiencies was not caused by noble metal contaminants.

**Figure 4:**
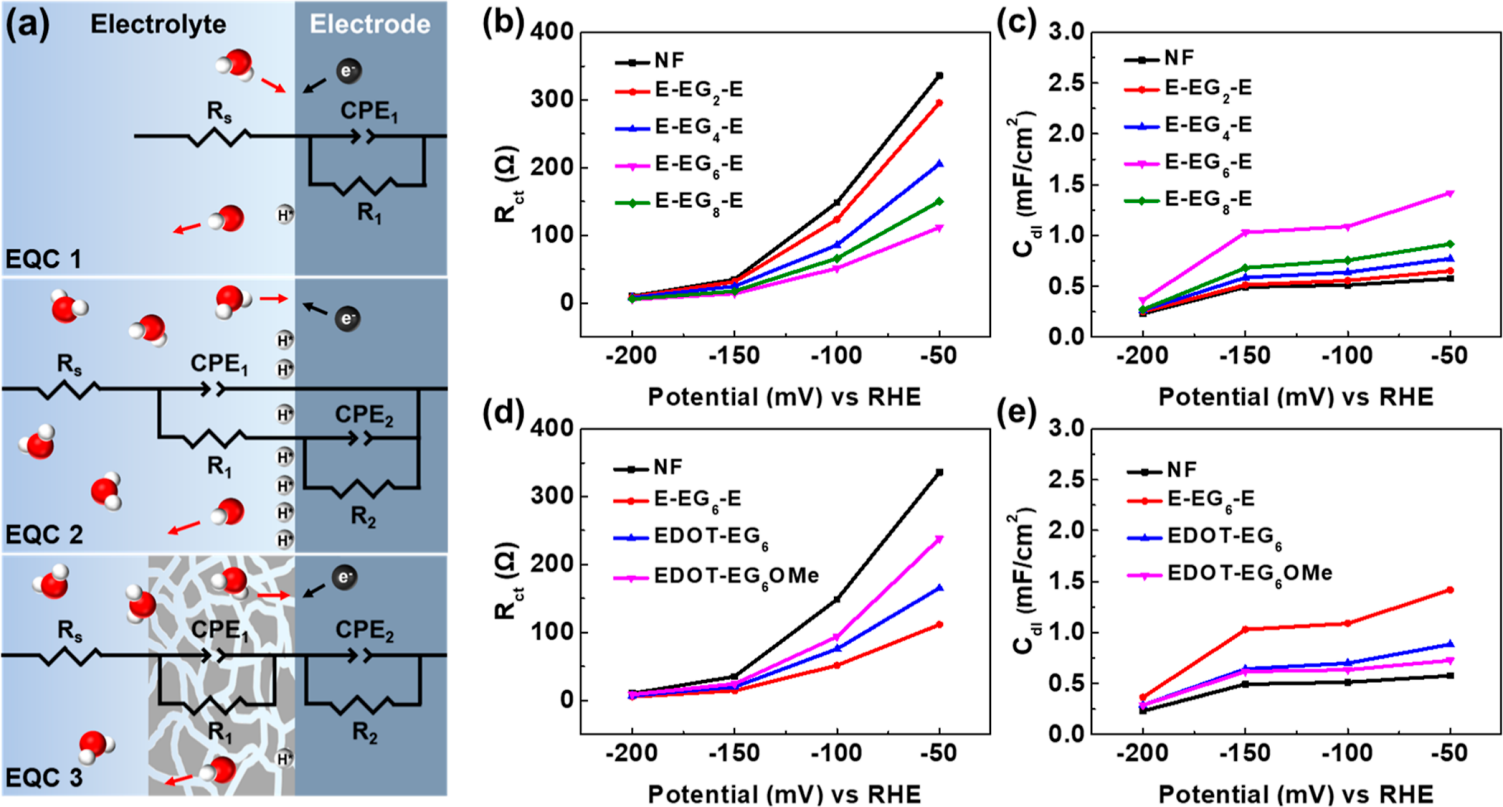
(a) Commonly used equivalent circuit models for HER and the illustration of electrode surface during HER process. (b) Fitted *R*_ct_ values of E-EG_*n*_-E. (c) Fitted *C*_dl_ values of E-EG_*n*_-E. (d) Fitted *R*_ct_ values of EG-functionalized EDOTs with 6EG groups. (e) Fitted *C*_dl_ values of EG-functionalized EDOTs with 6EG groups.

Next, we focused on investigating the surface cation concentrations of EG-functionalized EDOTs during the HER process. EIS experiments were applied to estimate the charge-transfer resistance (*R*_ct_) and the double-layer capacitance (*C*_dl_) under the applied potentials. For HER electrocatalysts, three types of equivalent circuit models are commonly used (Figure 4a), including the one-time constant model (EQC 1) and the two-time constant models (EQC 2 and EQC 3). Constant phase elements (CPEs) were employed to fit the *C*_dl_ values due to the nonideal capacitive behavior possibly caused by the surface heterogeneity or the mixed ion adsorption/diffusion kinetics.^50^ EQC 1 is applicable when only one time constant is presented in the Nyquist and Bode plots. In this case, the *R*_s_ value refers to the solution resistance, while *R*_1_ is associated with the charge-transfer resistance. The physical interpretations of the equivalent circuit models are illustrated in Figure 4a. *C*_dl_ values can be calculated from the CPE_1_. The calculation of *C*_dl_ from CPE is described in the Materials and Methods section. For the two time-constant models, EQC 2 and EQC 3 are commonly used depending on the different situations of the experiments. For the parallel model (EQC 2), the high-frequency and low-frequency parts have different physical interpretations. *R*_1_ and CPE_1_ correspond to the HER reaction kinetics, while *R*_2_ and CPE_2_ are associated with hydrogen adsorption.^51^ For the series model (EQC 3), the high-frequency part is associated with the electrode porosity, while the low-frequency part represents the reaction kinetics.^52^ The selection of equivalent circuit models must be based on the experimental data. Specifically, if only one time constant is presented in the Nyquist and Bode plots, then EQC 1 should be applied. On the other hand, EQC 2 and EQC 3 are applicable when two time constants are presented. Since the high-frequency parts of EQC 2 and EQC 3 correspond to the reaction kinetics and the electrode porosity, if the fitted *R*_1_ values are independent of the applied potentials, the series model should be used. On the contrary, if *R*_1_ values decrease significantly with increasing potential, the parallel model should be applied.^51,52^

The Nyquist plots and the Bode plots of blank NF, E-EG_2_-E, E-EG_4_-E, E-EG_6_-E, E-EG_8_-E, EDOT-EG_6_, and EDOT-EG_6_OMe are shown in Figures S21 and S22. The two semicircles in the Nyquist plots were almost overlapping (Figure S23). The existence of two overlapping peaks in the phase angles of Bode plots clearly indicated that the two-time-constant model should be applied. The fitted *R*_1_ values were almost independent of the applied potential, while *R*_2_ values decreased with increasing potential. Therefore, the series model (EQC 3) was applied to fit the experimental data. Note that EQC1 was applied at high overpotential since only one time constant was presented in the Bode plots.

The fitted *R*_2_ values corresponded to the charge-transfer resistance *R*_ct_, and CPE_2_ was used for the calculation of *C*_dl_. The fitted *R*_ct_ and *C*_dl_ values under different applied potentials are shown in Figure 4b–e. From Figure 4b, the highest *R*_ct_ values were observed on blank NF. The charge-transfer resistance decreased with increasing EG chain length, and the lowest *R*_ct_ was observed on E-EG_6_-E. Slightly higher *R*_ct_ values were observed on E-EG_8_-E. The trend in *R*_ct_ values was consistent with the reaction overpotentials observed in LSV measurements. Similarly, the highest *C*_dl_ value was observed on E-EG_6_-E (Figure 4c), indicating the higher ion concentration near the electrode surface under applied potentials. Lower *C*_dl_ values were observed on E-EG_8_-E, E-EG_4_-E, and E-EG_2_-E, and the lowest *C*_dl_ was observed on blank NF. To compare the effect of different molecular structures, the fitted *R*_ct_ and *C*_dl_ values of E-EG_6_-E, EDOT-EG_6_, and EDOT-EG_6_OMe are presented in Figure 4d,e. The *R*_ct_ values of EDOT-EG_6_ were lower than those of EDOT-EG_6_OMe, while the lowest *R*_ct_ values were observed on E-EG_6_-E. *C*_dl_ values decreased in the order E-EG_6_-E > EDOT-EG_6_ > EDOT-EG_6_OMe. The experimental results were also consistent with the reaction overpotentials observed in LSV measurements. The electrodes with lower *R*_ct_ and higher *C*_dl_ values demonstrated lower reaction overpotentials and better HER efficiency. The high consistency between the EIS and LSV measurements manifested the relation between the surface ion concentration and HER efficiency.

To further verify the increase in surface ion concentration under applied potentials, EQCM-D measurements were conducted on blank NF, E-EG_2_-E, E-EG_4_-E, E-EG_6_-E, E-EG_8_-E, EDOT-EG_6_, and EDOT-EG_6_OMe electrodes. QCM-D is a highly sensitive technique to monitor the subtle changes in the mass loading and viscoelastic properties of the solid–liquid interface.^53,54^ The shift in frequency values (Δ*f*) from QCM-D measurements can be correlated with the mass change on the surface.^55^ When the quartz crystal sensor was immersed in the solution, it oscillated at a specific resonant frequency (*f*_0_). As the ions or molecules are adsorbed on the surface, the resonant frequency is lowered to a different value (*f*). QCM-D measures the shift in the resonant frequency at different overtones, and the frequency shift Δ*f* = *f* – *f*_0_*i*s recorded. On the contrary, if the ions or molecules are leaving away from the surface, the resonant frequency will be increased. In addition, QCM-D can monitor the change of viscoelastic properties of the materials by measuring the energy dissipation factor (*D*), which is defined as , where *E*_d_ is the loss modulus and *E*_s_ is the storage modulus. QCM-D measures the change in the dissipation factor Δ*D* = *D* – *D*_0_, where *D*_0_ is the dissipation factor of the QCM sensor immersed in the solution, and *D* is the dissipation factor when materials or ions are absorbed on the surface. The increase in dissipation factor indicates a faster energy decay in the quartz crystal, which is generally due to the adsorption of ions/molecules or the swelling of materials.^56,57^ Moreover, EQCM-D measurements can be performed by integrating the QCM-D system with a potentiostat.

In this study, EQCM-D measurements were conducted in 0.01 M KOH. The lower concentration compared to the electrolyte solution used in electrochemical measurements was to avoid damage to the flow cell. Open circuit potential (OCP) of the system was first measured, and a potential of 0 V vs OCP was applied to the sensor. All the applied potentials reported in the EQCM-D experiments were referenced to the OCP of the system. The EQCM-D measurements started after a stable baseline had been reached. For the first 2 min of measurements, the applied potential was 0 V. The potential was increased to −25, −50, −75, and −100 mV during 2–12, 12–22, 22–32, and 32–42 min, respectively. Finally, the potential was turned back to 0 V and held for 10 min until the end of the measurement. The maximum potential applied in EQCM-D experiments was −100 mV, which was much smaller than the potential applied in the LSV experiments. The low potential values were selected to avoid damaging the QCM sensors and to minimize the fluctuations caused by bubble formation.

**Figure 5:**
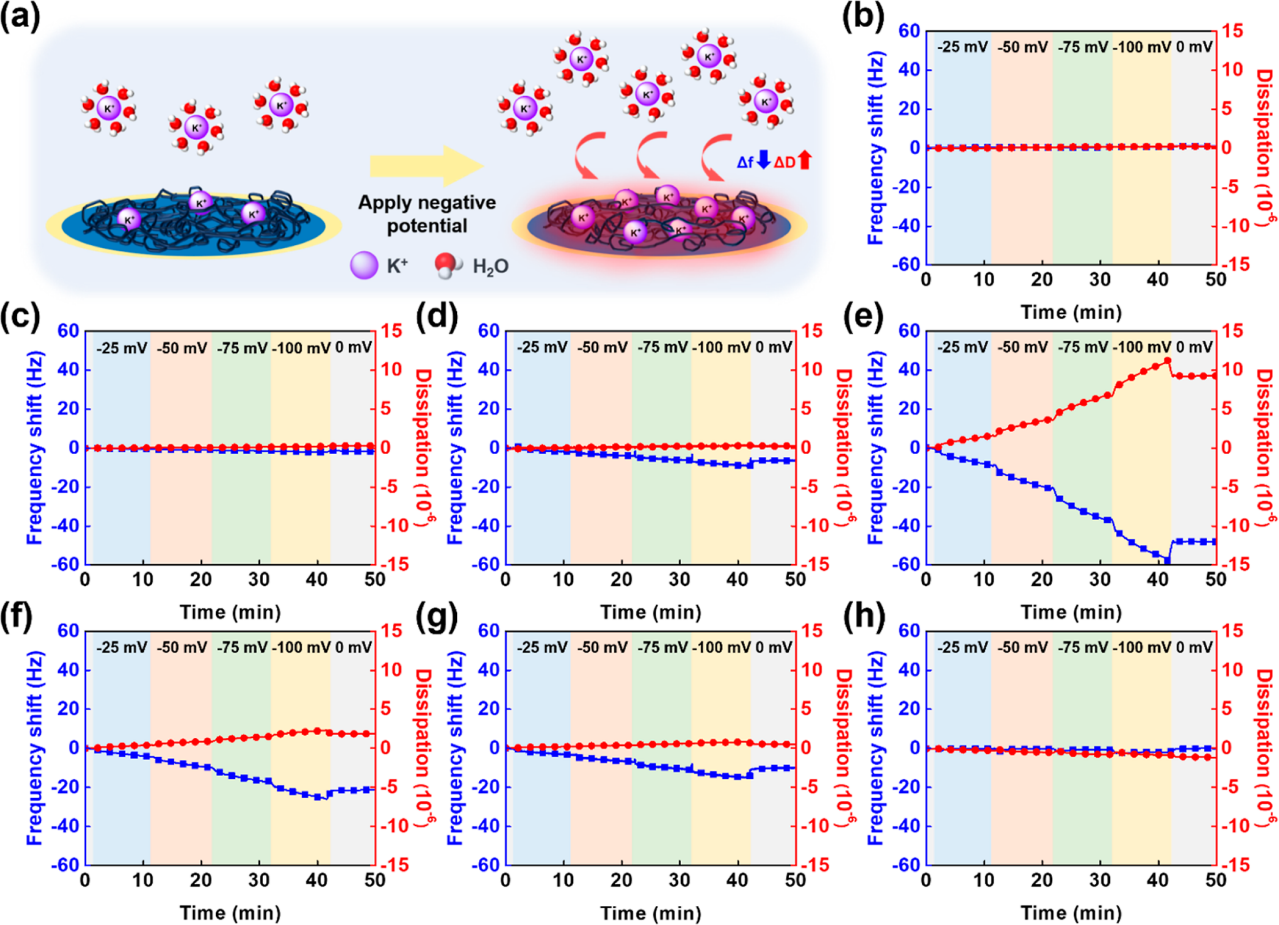
(a) Illustration of the QCM sensor surface during EQCM-D measurements. The real-time Δ*f* and Δ*D* of (b) bare Au, (c) E-EG_2_-E, (d) E-EG_4_-E, (e) E-EG_6_-E, (f) E-EG_8_-E, (g) EDOT-EG_6_, and (h) EDOT-EG_6_OMe.

The real-time Δ*f* and Δ*D* of the EQCM-D measurements are shown in Figure 5b–h, and the Δ*f* values at 40 min are presented in Figure S24. From Figure 5b, little change was observed when negative potentials were applied on bare Au. When E-EG_*n*_-E was present, the QCM sensors became sensitive to the applied potentials (Figure 5c–f). As the potential decreased, Δ*f* shifted to more negative values, indicating the increased ion adsorption on the polymer films, which is illustrated in Figure 5a. Previous studies have shown that PEDOT films can act as anion exchangers when PEDOT is electropolymerized in the presence of relatively small anions such as ClO_4_^–^ or NO_3_^–^. The movement of anions was observed during the redox process of the polymer films.^58,59^ In this study, the electrodes were immersed in a KOH solution until a stable baseline was reached before the beginning of EQCM-D measurements. The ClO_4_^–^ counterions in the polymer films were supposed to be replaced by OH^–^ anions. The repelling of OH^–^ anions was possible due to the negative potentials. Nevertheless, the repelling of anions would result in an increased resonant frequency due to the reduced mass on the electrodes. The decrease in the resonant frequency in Figure 5 cannot be solely explained by the movement of OH^–^ anions. Therefore, we suggested that the decreased Δ*f* was attributed to the increased K^+^ ion concentration on the negatively polarized electrode surface. From Figure S24, the Δ*f* at 40 min were −1.88, −8.77, −54.89, and −24.92 Hz for E-EG_2_-E, E-EG_4_-E, E-EG_6_-E, and E-EG_8_-E, respectively. The largest decrease in Δ*f* was observed on E-EG_6_-E, indicating the highest amount of K^+^ ions on the polymer film. The decrease in resonant frequency was smaller for E-EG_8_-E, corresponding to the slightly higher reaction overpotential compared to E-EG_6_-E. In contrast, only minor reductions in Δ*f* can be seen on E-EG_2_-E and E-EG_4_-E. In addition, large Δ*D* were observed on E-EG_6_-E and E-EG_8_-E, which demonstrated the swelling of polymer films due to the large amount of ion adsorption.^60^ The results of the EQCM-D experiments were consistent with the higher *C*_dl_ values observed in EIS measurements. To compare the effect of different molecular structures, the real-time Δ*f* and Δ*D* of EDOT-EG_6_ and EDOT-EG_6_OMe are shown in Figure 5g,h. The Δ*f* at 40 min were −14.45 Hz for EDOT-EG_6_ and −2.12 Hz for EDOT-EG_6_OMe. The lower Δ*f* for EDOT-EG_6_ represented a higher extent of ion adsorption, which was also consistent with the higher *C*_dl_ values in EIS measurements. In summary, the results in the EQCM-D experiments further confirmed the capability of EG-functionalized EDOTs to enhance the surface ion concentration under applied potentials.

XPS measurements were also conducted to estimate the relative amount of the potassium element on the electrode surface under applied potential. E-EG_2_-E, E-EG_4_-E, E-EG_6_-E, E-EG_8_-E, EDOT-EG_6_, and EDOT-EG_6_OMe were electropolymerized on Au substrates and were immersed in 1 M KOH. A −200 mV [vs reversible hydrogen electrode (RHE)] charge was applied on the electrodes for 2 min. The electrodes were taken out of the solution while keeping the applied voltage. After drying at 80 °C on a hot plate, XPS analysis was carried out to evaluate the amount of potassium on the electrodes. The K 2p XPS spectra are shown in Figure S25. The areas under the curves of XPS spectra were used to estimate the relative amount of potassium on the electrodes. The highest area was observed on E-EG_6_-E, indicating the highest amount of potassium element remaining on the electrode. The calculated areas under XPS spectra for E-EG_2_-E, E-EG_4_-E, E-EG_8_-E, EDOT-EG_6_, and EDOT-EG_6_OMe were divided by the area of E-EG_6_-E to compare the relative potassium amount on the electrodes (Table S2). The results in Table S2 suggested that the amount of potassium on E-EG_*n*_-E increased with increasing EG chain length, and a greater amount of potassium was observed on E-EG_6_-E than on EDOT-EG_6_ and EDOT-EG_6_OMe. The XPS results were highly consistent with the EQCM-D experiments and further emphasized the ability of EG-functionalized EDOTs to enhance the surface ion concentration under negative polarization.

### Effect of Surface Ion Concentration on Water Dissociation

The above experiments manifested the higher surface ion concentrations induced by EG-functionalized EDOTs. Previous studies have shown that the locally concentrated alkali metal cations can facilitate alkaline HER by stabilizing the water dissociation transition state and the products of water dissociation.^61^ Other publications have proposed that the concentrated K^+^ near the negatively polarized electrode surface can facilitate the water dissociation step by the *H–OH^δ−^–K^+^ interaction.^14^ In addition, recent studies have demonstrated that the conducting polymers could increase the concentration of hydrogen ions by regulating the local pH on the surface of the catalysts, promoting the activation of water molecules.^62,63^ Tafel slope is an important parameter for analyzing the HER mechanism.^64,65^ Generally, alkaline HER was considered to occur in two types of mechanisms. The first step is the Volmer step (H_2_O + e^–^ → H_ads_ + OH^–^), followed by the Heyrovsky step (H_ads_ + H_2_O + e^–^ → H_2_ + OH^–^). The second mechanism involves the Volmer step followed by the Tafel step (H_ads_ + H_ads_ → H_2_).^66,67^ For HER with the Volmer, Heyrovsky, and Tafel rate-determining steps, Tafel slopes of 120, 40, and 30 mV dec^–1^ are expected.^68^

**Figure 6:**
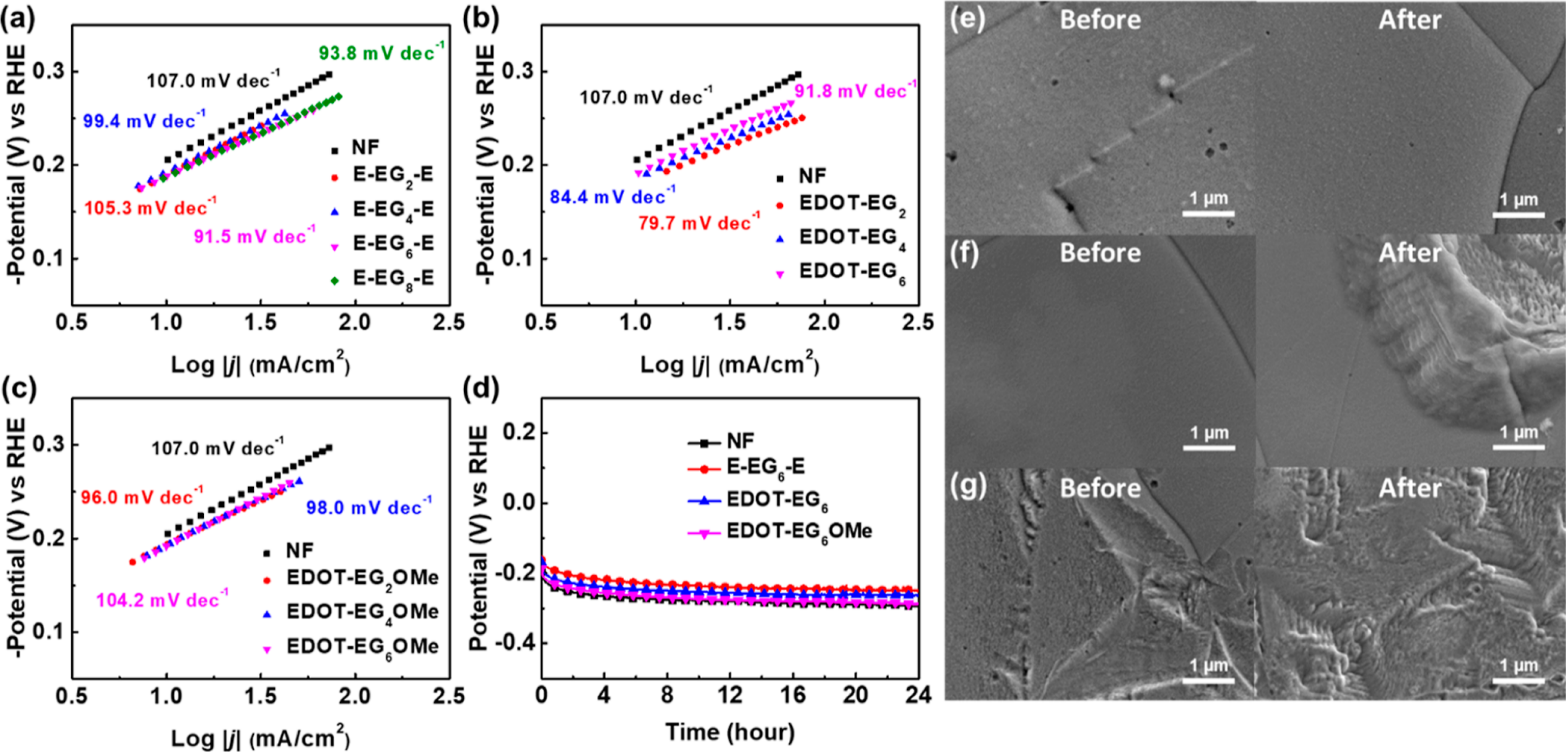
Tafel plots of (a) E-EG_*n*_-E, (b) EDOT-EG_*n*_, and (c) EDOT-EG_*n*_OMe. (d) Chronopotentiometry measurements of EG-functionalized EDOTs with 6EG groups. SEM images before (left) and after (right) 24 h of reaction for (e) E-EG_6_-E, (f) EDOT-EG_6_, and (g) EDOT-EG_6_OMe.

The Tafel plots of EG-functionalized EDOTs are shown in Figure 6a-c. The highest Tafel slope of 107.0 mV dec^–1^ was observed on the blank NF, indicating the sluggish water dissociation (Volmer step) kinetics without polymer coatings. After introducing EG-functionalized EDOTs, the Tafel slopes were decreased. For E-EG_*n*_-E, the Tafel slope decreased with increasing EG chain length. The lowest Tafel slope was observed on E-EG_6_-E, and the Tafel slope of E-EG_8_-E was slightly increased in accordance with the HER efficiencies in the LSV measurements. On the contrary, higher Tafel slopes were observed on EDOT-EG_*n*_ and EDOT-EG_*n*_OMe with longer EG chains. The trends in the Tafel slopes were consistent with those in the reaction overpotentials from LSV measurements. The reduced Tafel slopes corresponded to the accelerated water dissociation kinetics.^69^ Combining the results of Tafel plots with EIS, EQCM-D, and XPS measurements, we concluded that the EG-functionalized EDOTs could induce concentrated potassium ions near the electrode surface under negative polarization. The locally concentrated ions can stretch the H–OH bonding through the noncovalent interaction between potassium ions and water molecules. The *H–OH^δ−^–K^+^ interaction facilitated the rate-determining water dissociation step and, therefore, improved the alkaline HER.

To further confirm the positive role of K^+^ in facilitating HER, we measured the HER performance of E-EG_6_-E, EDOT-EG_6_, and EDOT-EG_6_OMe in the organic cationic system containing tetramethylammonium (TMA^+^). The LSV curves are shown in Figure S26. The HER efficiencies were decreased in the presence of TMA^+^ after coating NF with EG-functionalized EDOTs, which manifested the crucial role of potassium ions in promoting alkaline HER efficiency.

### Stability

The electrochemical stability is important for practical applications of HER electrocatalysts. Chronopotentiometry tests were performed at 10 mA cm^–2^ for 24 h to evaluate the stability of the electrodes. The results are listed in Figure 6d. For blank NF, the potential required to achieve 10 mA cm^–2^ shifted to a more negative value by 98 mV after 24 h of reaction. The inferior HER stability of NF can be ascribed to the transformation of nickel to nickel hydroxide during the alkaline HER.^70^ After NF were coated with E-EG_6_-E, EDOT-EG_6_, and EDOT-EG_6_OMe and subjected to 24 h of reaction, the reaction overpotentials were increased by 87, 96, and 101 mV, respectively. Since nickel served as the electrocatalyst, poor HER stability was expected. The SEM images before and after the chronopotentiometry measurements are shown in Figure 6e–g. From Figure 6e, the surface morphology of E-EG_6_-E remained almost unchanged after 24 h of reaction, which can be attributed to the chemical cross-linking that enhanced the film stability.^71,72^ For EDOT-EG_6_ and EDOT-EG_6_OMe, a significant change in surface morphologies can be observed in Figure 6f,g. The transformation of surface morphologies was due to the continuous evolution of gas bubbles that led to the damage of polymer films.^73^ The damaged polymer films lost the functionality to induce locally concentrated ions and may instead block the reaction active sites from the electrolytes. As a consequence, similar or higher reaction overpotentials were required on EDOT-EG_6_ and EDOT-EG_6_OMe compared to blank NF after 24 h of reaction. The superior electrode stability of E-EG_6_-E emphasized the importance of molecular structure design for electrode modification.

## Conclusions

In this study, we successfully synthesized EG-functionalized EDOTs with different molecular structures and different numbers of EG moieties. The synthesized monomers were electropolymerized on the NF electrodes. LSV measurements were performed in 1 M KOH to evaluate the HER performance of the electrodes. The HER efficiencies were enhanced after the introduction of EG-functionalized EDOTs. The effect of the EG chain length was also investigated. Decreasing reaction overpotentials with increasing EG chain length were observed on E-EG_*n*_-E. Conversely, for EDOT-EG_*n*_ and EDOT-EG_*n*_OMe, higher reaction overpotentials were required to reach the same level of current density on molecules with longer EG chains. The opposite trend between the HER efficiency and EG chain length was ascribed to the inferior film-forming ability of EDOT-EG_*n*_ and EDOT-EG_*n*_OMe. LSV results for polymers with similar structures but without EG moieties (E-C_12_-E and EDOT-C_12_) demonstrated the importance of EG functional groups in promoting alkaline HER. By replacing the Pt counter electrode with a GC electrode, we confirmed that the improved HER efficiency was not caused by the dissolving noble metal contaminants. Water contact angle measurements were conducted for all polymers on Au substrate. Reduced contact angles were observed, indicating better hydrophilicity of the conducting polymer films. However, LSV results of polymers with excellent hydrophilicity (EDOT-S and EDOT-PC) manifested that the hydrophilicity was not the dominating factor for the improved HER efficiency.

EIS, EQCM-D, and XPS analyses were employed to investigate the origin of the enhanced HER performance. From the EIS fitting results, higher *C*_dl_ and lower *R*_ct_ values were observed on the electrodes with better HER efficiency. In addition, a greater decrease in Δ*f* was seen on the electrodes with lower reaction overpotentials when negative potentials were applied in the EQCM-D measurements. Calculated area ratios from the XPS spectra revealed a greater amount of potassium remaining on the E-EG_6_-E surface. Combining the results from EIS, EQCM-D, and XPS measurements, we suggested that locally concentrated potassium ions were induced when negative potentials were applied to the electrodes coated with EG-functionalized EDOTs. The locally concentrated ions could facilitate the water dissociation step by the *H–OH^δ−^–K^+^ interaction, as evident from the reduced Tafel slopes. Finally, chronopotentiometry tests were conducted in a 1 M KOH solution. Improved electrode stability was observed on E-EG_6_-E compared to that of blank NF. Comparable and reduced stabilities were seen on EDOT-EG_6_ and EDOT-EG_6_OMe, respectively. From the SEM images after the chronopotentiometry tests, significant changes in surface morphologies were observed on EDOT-EG_6_ and EDOT-EG_6_OMe, while the surface of E-EG_6_-E remained almost unchanged. The improved stability of E-EG_6_-E can be attributed to the chemical cross-linking in the polymer film. The damaged polymer films could block the reaction active sites from the electrolytes, leading to reduced electrode stability. The present study highlighted the importance of molecular structures and functionalities on electrode surface modification. The efficiency and stability of electrocatalysts can be improved simultaneously with the rational design of materials.

## Materials and Methods

### Chemicals

Acetonitrile (ACN), potassium hydroxide (KOH), hydroxymethyl EDOT (EDOT–OH), and 25% tetramethylammonium hydroxide (TMAH) aqueous solution were purchased from Sigma-Aldrich. Dichloromethane (DCM) was purchased from Fisher Scientific. Sodium dodecyl sulfate (SDS) was purchased from ACROS. Lithium perchlorate (LiClO_4_) was purchased from Alfa Aesar. Tetrabutylammonium perchlorate (TBAP) was purchased from Tokyo Chemical Industry Co., ltd. Ethanol was purchased from Echo Chemical Co., Ltd. All of the chemicals were used without further purification.

### Synthesis of Functionalized EDOTs

Synthesis of functionalized EDOTs was based on similar published synthetic processes.^38,74,75^ All reactions were performed using standard vacuum-line and Schlenk techniques.

For the synthesis of E-EG_2_-E, E-EG_4_-E, E-EG_6_-E, and E-EG_8_-E, oligo EGs (OEG) (27 mmol) and triethylamine (TEA) (54 mmol) were dissolved in 40 mL of CH_2_Cl_2_ and stirred at 0 °C. *p*-Toluenesulfonyl chloride (TsCl) (65 mmol) was dissolved in 40 mL of CH_2_Cl_2_. The TsCl solution was added dropwise into the solution containing OEG and TEA. The mixture was elevated to room temperature and reacted for 24 h under a N_2_ atmosphere. The reaction was quenched by water and stirred for 30 min. The mixture was extracted with saturated NaCl_(aq)_. The organic layer was collected and dried over MgSO_4_, and the solvent was removed under reduced pressure. The crude product was purified by column chromatography.

Next, the products from the previous step (4.65 mmol) were dissolved in 10 mL of dry DMF and added dropwise into the mixture of EDOT–OH (11.6 mmol), NaH (46.5 mmol), and 18-crown-6 (1.85 mmol) in 30 mL of dry DMF at 0 °C. The mixture was stirred at room temperature for 24 h under N_2_. The reaction was quenched by water and stirred for 30 min. The mixture was extracted with ethyl acetate. The organic layer was collected and dried over MgSO_4_, and the solvent was removed under reduced pressure. The crude product was purified by column chromatography to yield E-EG_2_-E, E-EG_4_-E, E-EG_6_-E, and E-EG_8_-E.

To synthesize E-C_12_-E, 1,12-dibromododecane (15.2 mmol) was dissolved in 15 mL of dry tetrahydrofuran (THF) and added into the mixture of EDOT–OH (38 mmol), NaH (152 mmol), and 18-crown-6 (6.08 mmol) in 40 mL of dry DMF at 0 °C. The mixture was stirred at room temperature for 24 h under a N_2_ atmosphere. The reaction was quenched by water and stirred for 30 min. The mixture was extracted with ethyl acetate. The organic layer was collected and dried over MgSO_4_, and the solvent was removed under reduced pressure. The crude product was purified by column chromatography to yield a white solid.

For EDOT-C_12_, EDOT–OH (29 mmol), NaH (145 mmol), and 18-crown-6 (2.9 mmol) were dissolved in 70 mL of anhydrous THF and stirred at 0 °C. 1-Bromododecane (34.8 mmol) was added dropwise. The mixture was stirred at room temperature for 24 h under N_2_. The product was diluted with 200 mL of NH_4_Cl aqueous solution and extracted with CH_2_Cl_2_. The organic phase was collected and dried with MgSO_4_, and the solvent was removed under reduced pressure. The crude product was purified by column chromatography to yield a colorless oil.

For EDOT-EG_2_OMe, EDOT-EG_4_OMe, and EDOT-EG_6_OMe, methyl-EG_*n*_-bromide (*n* = 2, 4, 6) (5.5 mmol) was dissolved in 10 mL of dry THF and added into the mixture of EDOT–OH (5 mmol), NaH (25 mmol), and 18-crown-6 (1 mmol) in 20 mL of dry DMF at 0 °C. The mixture was stirred at room temperature for 24 h under N_2_. The reaction was quenched by water and stirred for 30 min. The product was extracted with CH_2_Cl_2_. The organic layer was dried over MgSO_4_, and the solvent was removed under reduced pressure. The crude product was purified by column chromatography.

For EDOT-EG_2_, EDOT-EG_4_, and EDOT-EG_6_, OEG (200 mmol) and trityl chloride (40 mmol) were dissolved in 40 mL of CH_2_Cl_2_. A 40 mmol portion of pyridine was added into the solution, and the mixture was stirred at room temperature for 24 h under N_2_. The product was extracted with CH_2_Cl_2_. The organic layer was collected and dried over MgSO_4_, and the solvent was removed under reduced pressure. The crude product was purified by column chromatography.

Next, the products from the first step (42.5 mmol) were dissolved in 75 mL of CH_2_Cl_2_. TEA (77 mmol) was added, and the mixture was cooled to 0 °C. Methanesulfonyl chloride (63.75 mmol) was added dropwise. The mixture was elevated to room temperature and stirred for 24 h under nitrogen. The reaction was quenched by water and stirred for 30 min. The mixture was extracted with CH_2_Cl_2_. The organic layer was dried over MgSO_4_, and the solvent was removed under reduced pressure. The crude product was purified by column chromatography.

Finally, the products from the second step (15 mmol) were dissolved in 10 mL of dry DMF and added into 40 mL of DMF solution containing NaH (50 mmol), 18-crown-6 (2 mmol), and EDOT–OH (10 mmol). The mixture was stirred at room temperature for 24 h under N_2_. The reaction was quenched by water and stirred for 30 min. The product was extracted with ethyl acetate; the organic layer was dried over MgSO_4_, and the solvent was removed under reduced pressure. The crude product was added into the mixture of 100 mL of methanol and Amberlite IR-120 (12.18 g). The mixture was stirred at 60 °C for 6 h under nitrogen, Amberlite IR-120 was filtered, and the solvent was removed under reduced pressure. The product was purified by column chromatography to yield EDOT-EG_2_, EDOT-EG_4_, and EDOT-EG_6_.

The chemical structures and ^1^H NMR and ^13^C NMR spectra of E-EG_8_-E and E-C_12_-E are provided in Supporting Information.

### Electropolymerization

All of the experiments were performed using an Autolab PGSTAT128N potentiostat (Metrohm, Netherlands) in a three-electrode system containing a platinum counter electrode. Ag/AgCl (saturated KCl) and Ag/Ag^+^ reference electrodes were used in aqueous and organic solutions, respectively. NF was used as the working electrode. Prior to use, all of the NF electrodes were cleaned with 1 M HCl, deionized (DI) water, and ethanol and were dried at 80 °C.

A thin layer of EDOT–OH was predeposited on NF for better adhesion between the polymer film and the substrate.^38^ The electropolymerization of EDOT–OH was carried out in an aqueous solution containing 10 mM EDOT–OH, 50 mM SDS, and 100 mM LiClO_4_ by applying 1 cycle of potential scan from −0.6 to 1.1 V versus Ag/AgCl. Subsequently, the electrode was rinsed with DI water and dried with N_2_ to remove the excess electrolytes.

For the preparation of monomer solutions, 10 mM E-EG_2_-E, E-EG_4_-E, E-EG_6_-E, E-EG_8_-E, EDOT-EG_2_, EDOT-EG_4_, EDOT-EG_6_, EDOT-EG_2_OMe, EDOT-EG_4_OMe, EDOT-EG_6_OMe, and EDOT-C_12_ were dissolved in ACN in the presence of 100 mM LiClO_4_. For E-C_12_-E, 10 mM monomer was dissolved in DCM containing 100 mM TBAP.

The electropolymerization of E-EG_2_-E, E-EG_4_-E, E-EG_6_-E, and E-EG_8_-E was performed by applying 3 cycles of potential scan from −0.6 to 1.1 V versus Ag/Ag^+^. For EDOT-EG_2_, EDOT-EG_4_, EDOT-EG_6_, EDOT-EG_2_OMe, EDOT-EG_4_OMe, EDOT-EG_6_OMe, E-C_12_-E, and EDOT-C_12_, 3 cycles of potential scan from −0.6 to 1.4 V versus Ag/Ag^+^ were applied. After the electropolymerization process, the electrodes were rinsed with ACN and DI water and dried with N_2_ to remove excess electrolytes or any soluble products. The NF electrodes coated with poly(E-EG_2_-E), poly(E-EG_4_-E), poly(E-EG_6_-E), poly(E-EG_8_-E), poly(EDOT-EG_2_), poly(EDOT-EG_4_), poly(EDOT-EG_6_), poly(EDOT-EG_2_OMe), poly(EDOT-EG_4_OMe), poly(EDOT-EG_6_OMe), poly(E-C_12_-E), and poly(EDOT-C_12_) were denoted as E-EG_2_-E, E-EG_4_-E, E-EG_6_-E, E-EG_8_-E, EDOT-EG_2_, EDOT-EG_4_, EDOT-EG_6_, EDOT-EG_2_OMe, EDOT-EG_4_OMe, EDOT-EG_6_OMe, E-C_12_-E, and EDOT-C_12_, respectively.

The copolymerization of EDOT-S and EDOT-PC with E-EG_6_-E was conducted in an aqueous solution containing 5 mM EDOT-S/EDOT-PC and 5 mM E– EG_6_-E. 50 mM SDS (50 mM) was added as a surfactant, and 100 mM LiClO_4_ served as an electrolyte. Three cycles of potential scan from −0.6 to 1.1 V versus Ag/AgCl were applied. The electrodes were rinsed with DI water and dried with N_2_ after the electropolymerization process. The NF electrodes coated with poly(EDOT-S) and poly(EDOT-PC) were denoted as EDOT-S and EDOT-PC, respectively. The NF electrode coated with the copolymer of E-EG_6_-E and EDOT-S was denoted as E-EG_6_-E-*co*-EDOT-S, and the electrode coated with the copolymer of E-EG_6_-E and EDOT-PC was denoted as E–6-E-*co*-EDOT-PC.

### Characterization

The water contact angle measurements were performed by using a goniometer (Sindatek, Taiwan). All measurements were taken three times (*n* = 3) for the calculation of mean values and standard deviations. SEM was conducted by using a scanning electron microscope (Jeol, Japan). The accelerating voltage was 5 keV. Nuclear magnetic resonance (NMR) spectra were recorded on a Bruker AVIII HD 500 MHz NMR (^1^H 500 MHz, ^13^C 100 MHz) spectrometer. XPS data were recorded on a ULVAC-PHI (Quantes) XPS instrument with a dual scanning X-ray source (a hard X-ray source (Cr Kα) and a soft X-ray source [Al Kα]). 0.5 × 0.5 cm^2^ Au were used as substrates for the XPS measurements. Raman spectroscopy was conducted using a homemade apparatus. Laser light from a laser source (532 nm, WITec) was directed through a custom-built path onto the sample. Reflected light was then directed into a spectrometer (Kymera-328i, Andor) equipped with a cooling camera (DU420A-BEX2-DD, Andor).

### Electrochemical Measurements

All of the electrochemical measurements were performed with an Autolab PGSTAT128N potentiostat (Metrohm, Netherlands). The three-electrode system comprised a platinum counter electrode and a Ag/AgCl (saturated KCl solution) reference electrode. The experiments were conducted in a 1 M KOH aqueous solution. The potentials reported in this study were all referenced to a RHE according to the Nernst equation (*E*_RHE_ = *E*_Ag/AgCl_ + 0.197 + 0.0591 pH) unless otherwise specified. For LSV measurements in the organic cationic system, 2.5% TMAH aqueous solution (pH 13.6) was prepared by diluting 25% TMAH with DI water.

LSV measurements were conducted at a scan rate of 2 mV s^–1^. Before each measurement, multiple LSV scans were performed at a higher scan rate (5 mV s^–1^) in the same potential range until stable currents were obtained to ensure the catalysts were fully activated. 95% *iR* compensation was applied to the LSV data on the basis of EIS measurements. All of the LSV measurements were repeated 3 times to ensure the reproducibility of the experiments. The mean values and standard deviations of η_50_ were calculated from the repeated LSV curves.

For ECSA measurements, *C*_dl_ values were determined by performing CV at multiple scan rates (10, 25, 50, and 100 mV s^–1^) in the nonfaradaic region between a 0.1 V potential window centered at OCP. The *C*_dl_ values were calculated from the slope in the current density vs scan rate plot. ECSA was determined by dividing *C*_dl_ with the specific capacitance value of 0.04 mF cm^–2^.^76^

EIS experiments were performed with a frequency ranging from 100 kHz to 0.1 Hz. The EIS data were fitted by using Zview2 software. A [R(RQ)(RQ)] equivalent circuit was employed, where *R* represents resistors, and *Q* represents CPEs. CPEs are commonly used to model the behavior of an EDL.^51,77^ The impedance of a CPE (*Z*_CPE_) can be described as , where *Q* is a parameter containing the capacitance information and has the unit of F s^*n*–1^. *j* is the imaginary number, and ω is the angular frequency. *n* is a unitless parameter ranging from 0 to 1 that describes the deviation from ideal capacitive behavior.^50,78^ The effective capacitance (*C*_eff_) of a CPE can be calculated by , where *R* is the corresponding resistance in the parallel circuit.

Chronopotentiometry experiments were conducted at 10 mA cm^–2^ for 24 h to investigate the stability of the electrodes.

### EQCM-D Measurements

The EQCM-D measurements were performed using a QCM-D (QSense Explorer and Analyzer system) with a quartz crystal resonator (QSX 301 Au sensor). The fundamental resonance frequency of the QCM sensor was 5 MHz. A QEM 401 Q-Sense electrochemical module integrated with a PGSTAT204 potentiostat (Metrohm, Netherlands) was used to apply different potentials on the Au sensor. All experiments were performed at 25 °C. A three-electrode setup with a Ag/AgCl leak-free reference electrode (3.4 M KCl) was employed in the EQCM-D measurements. 0.01 M KOH solution was used as the electrolyte. A flow rate of 36.5 μL min^–1^ was controlled using a tubing pump.

Before each measurement, the OCP was measured and applied to the system under a constant flow of 0.01 M KOH. The EQCM-D measurements started after the frequency shift (Δ*f*) and the change in the energy dissipation factor (Δ*D*) reached equilibrium values. The EQCM-D data were recorded at five overtones (n = 1, 3, 5, 7, and 9) throughout the measurements. In this study, all of the frequency and dissipation values were taken from the third overtone (*n* = 3) since the first overtone was too sensitive that it may be influenced by any vibration, and the fifth to the ninth overtones could provide similar information.

All applied potentials reported in the EQCM-D experiments were referenced to the OCP of the system. 0 V was applied in the first 2 min of the measurements. The applied potential was increased to −25, −50, −75, and −100 mV during 2–12, 12–22, 22–32, and 32–42 min, respectively. Finally, the potential was switched to 0 V and held for 10 min until the end of the measurements.

## Supporting Information Available

^1^H NMR, ^13^C NMR, and HRMS ESI-MS spectra of E-EG_8_-E and E-C_12_-E; high-resolution SEM images; EDS elemental mapping; Raman spectra; repeated LSV curves; optical images of monomer solutions; LSV curves of E-EG_*n*_-E with different cycles of potential scan; current density vs scan rate plot; calculated ECSA; optical images of water droplets; water contact angles of NF, EDOT-S, EDOT-PC, E-C_12_-E, and EDOT-C_12_; LSV curves of E-C_12_-E and EDOT-C_12_; LSV curves with GC counter electrode; Nyquist plots; Bode plots; Δ*f* values at 40 min in EQCM-D measurements; K 2p XPS spectra; calculated area ratios from K 2p XPS spectra; and LSV curves in TMAH solutions (PDF)
