# Supplementary material for: Modulating Surface Cation Concentration via Tuning the Molecular Structures of Ethylene Glycol-Functionalized PEDOT for Improved Alkaline Hydrogen Evolution Reaction
Source: JACS Au. 2024 Jul 21;4(8):3070–83. doi: 10.1021/jacsau.4c00409 (PMC11350742; doi:10.1021/jacsau.4c00409)
Supplement: Supplementary file 1 — au4c00409_si_001.pdf [file au4c00409_si_001.pdf]

Supporting Information

Modulating Surface Cation Concentration via Tuning the Molecular Structures of Ethylene Glycol-Functionalized PEDOT for Improved Alkaline Hydrogen Evolution Reaction

*Hsun-Hao Lin, Hsuan-I Liang, and Shyh-Chyang Luo**

Department of Materials Science and Engineering, National Taiwan University, No. 1, Sec. 4, Roosevelt Road, Taipei, 10617, Taiwan.

*Corresponding author.

Email address: shyhchyang@ntu.edu.tw

Table of Contents

Figure S1: ^{1}H NMR spectrum of E-EG_8-E

Figure S2: ^{13}C NMR spectrum of E-EG_8-E

Figure S3. HRMS ESI-MS spectrum of E-EG_8-E

Figure S4: ^{1}H NMR spectrum of E-C_{12}-E

Figure S5: ^{13}C NMR spectrum of E-C_{12}-E

Figure S6. HRMS ESI-MS spectrum of E-C_{12}-E

Figure S7: SEM images of NF and poly(EDOT-OH)

Figure S8: High-resolution SEM images

Figure S9: EDS elemental mapping

Figure S10: Raman spectra

Figure S11: Repeated LSV curves

Figure S12: Optical images of solutions before and after electropolymerization

Figure S13: LSV results of E-EG_n-E with different cycles of potential scan

Figure S14: Current density vs scan rate plots

Table S1: Calculated ECSA values

Figure S15: Water contact angles on NF

Figure S16: Optical images of water droplets on EG-functionalized EDOTs

Figure S17: Water contact angles of EDOT-S and EDOT-PC

Figure S18: Water contact angles of E-C_{12}-E and EDOT-C_{12}

Figure S19: LSV curves of E-C_{12}-E and EDOT-C_{12}

Figure S20: LSV curves with GC counter electrode

Figure S21: Nyquist plots

Figure S22: Bode plots

Figure S23: Nyquist plots with smaller scales

Figure S24: Δf values in EQCM-D measurements at 40 min

Figure S25: K 2p XPS spectra

Table S2: Calculated area ratios from K 2p XPS spectra

Figure S26: LSV curves in TMAH solutions

E-EG$_8$-E:

^{1}H NMR (500 MHz, $CDCl_3$) δ (ppm) 6.28-6.3 (m, 4H), 4.27-4.31 (m, 2H), 4.22 (dd, J = 2.23, 11.65 Hz, 2H), 4.03 (dd, J = 7.49, 11.67 Hz, 2H), 3.72-3.76 (m, 2H), 3.6-3.67 (m, 34H) ^{13}C NMR (100 MHz, $CDCl_3$) δ (ppm) 141.55, 141.46, 99.63, 99.53, 72.59, 71.18, 70.62, 70.55, 70.53, 70.51, 69.60,66.10; HRMS (ESI-MS) m/z calcd for $C_{30}H_{46}O_{13}S_2$ [M^+]: 679.2453, found 679.2488.

E-C$_{12}$-E:

^{1}H NMR (500 MHz, $CDCl_3$) δ (ppm) 6.28-6.3 (m, 4H), 4.27-4.31 (m, 2H), 4.22 (dd, J = 2.24, 11.62 Hz, 2H), 4.03 (dd, J = 7.54, 11.63 Hz, 2H), 3.66 (dd, J = 5.00, 10.40 Hz, 2H), 3.57 (dd, J = 6.03, 10.40 Hz, 2H), 3.46 (t, J = 6.67 Hz, 4H), 1.2-1.3 (m, 20H) ^{13}C NMR (100 MHz, $CDCl_3$) δ (ppm) 141.60, 141.56, 99.65, 99.53, 72.64, 72.09, 69.08, 66.26, 29.55, 29.52, 29.42, 26.01; HRMS (ESI-MS) m/z calcd for $C_{26}H_{38}O_6S_2$ [M^+]: 511.2183, found 511.2185.

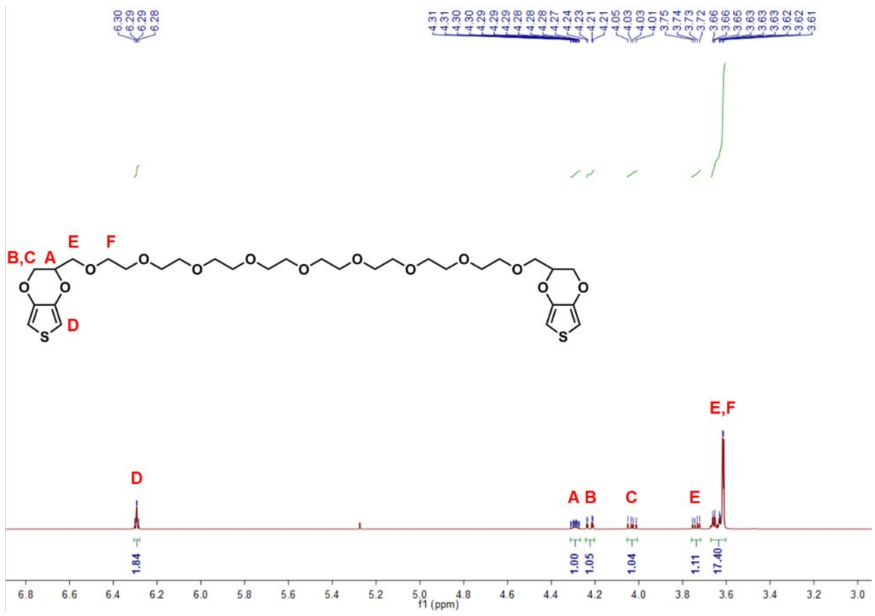

Figure S1. ^{1}H NMR spectrum of E-EG$_8$-E. (In $CDCl_3$, 500 MHz)

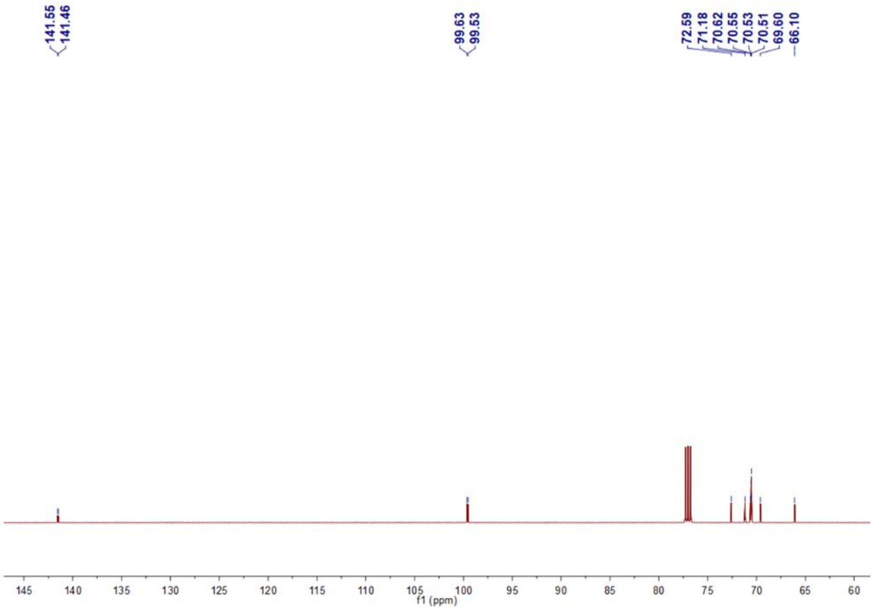

Figure S2. ^{13}C NMR spectrum of E-EG$_8$-E. (In $CDCl_3$, 100 MHz)

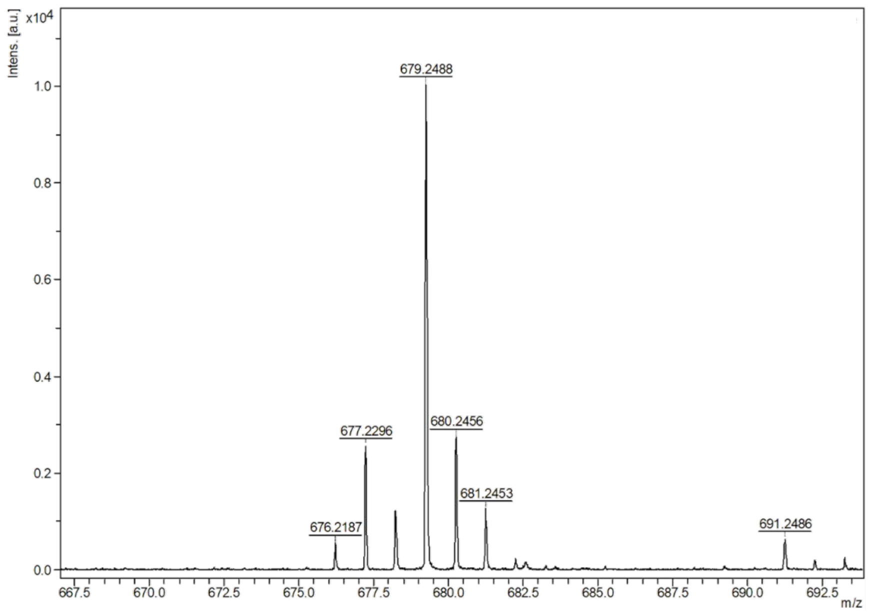

Figure S3. HRMS ESI-MS spectrum of E-EG$_8$-E

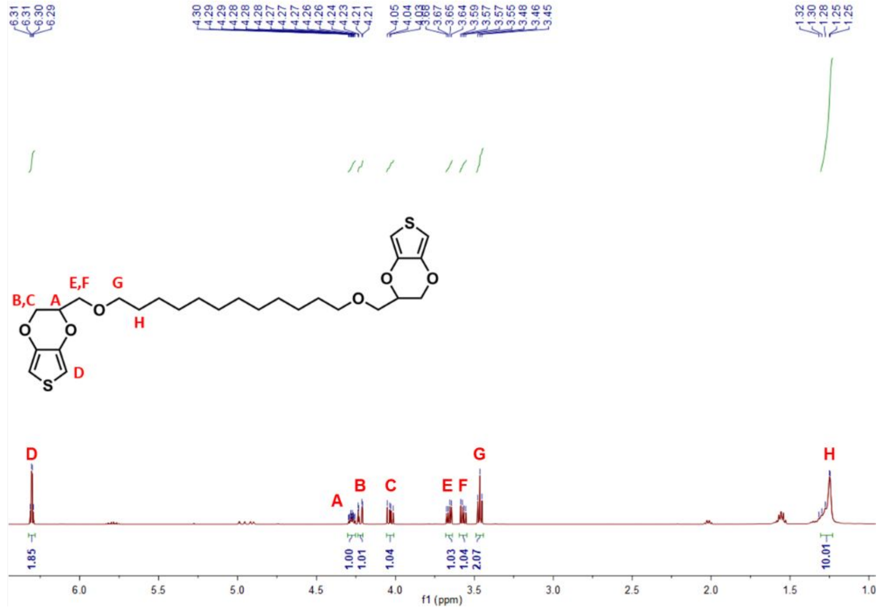

Figure S4. ^{1}H NMR spectrum of E-C$_{12}$-E. (In CDCl$_3$, 500 MHz)

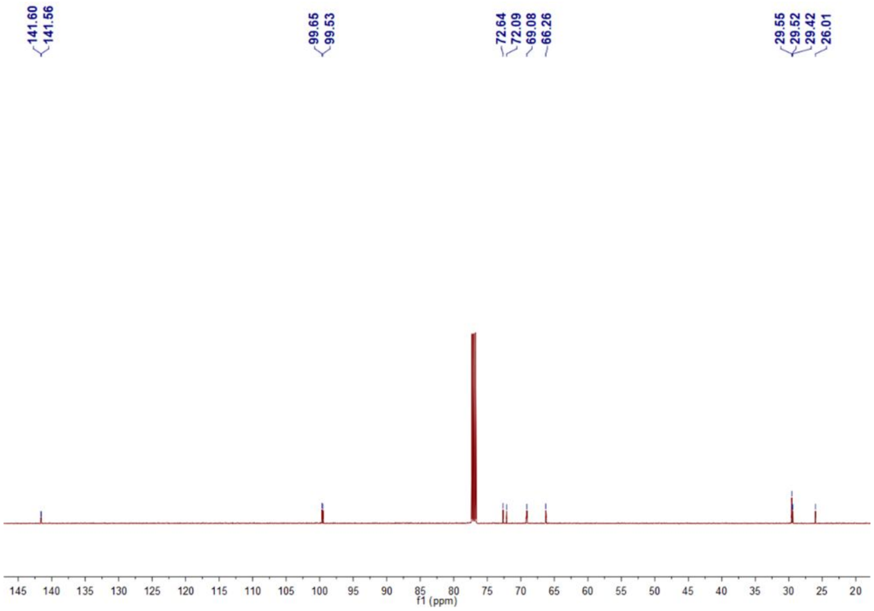

Figure S5. ^{13}C NMR spectrum of E-C_{12}-E. (In $CDCl_3$, 100 MHz)

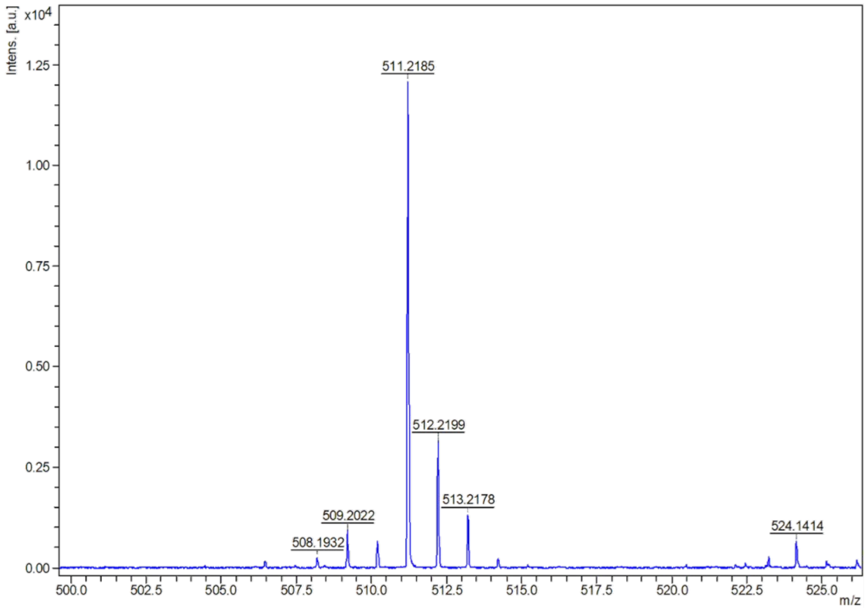

Figure S6. HRMS ESI-MS spectrum of E-C_{12}-E

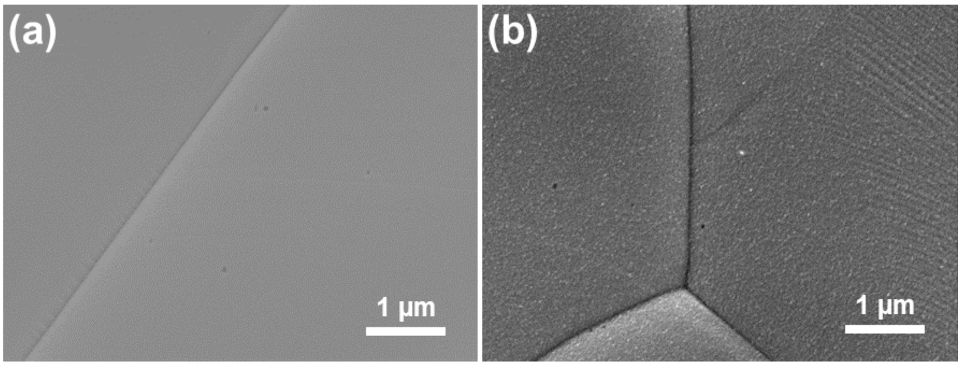

Figure S7. SEM images of (a) Blank NF and (b) poly(EDOT-OH).

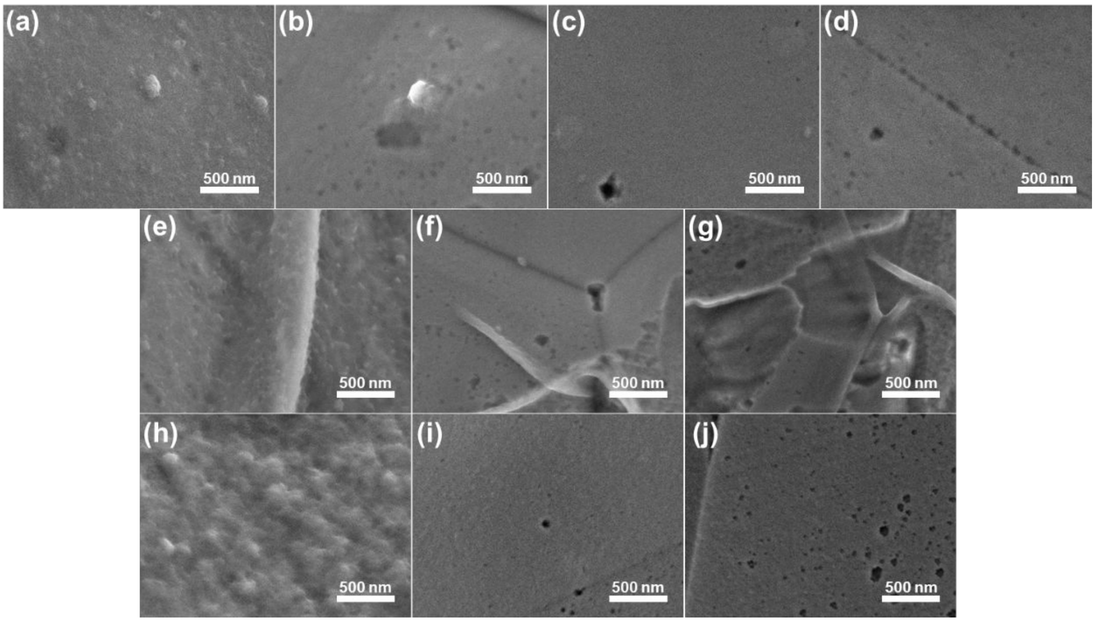

Figure S8. High-resolution SEM images of (a) E-EG$_2$-E, (b) E-EG$_4$-E, (c) E-EG$_6$-E, (d) E-EG$_8$-E, (e) EDOT-EG$_2$, (f) EDOT-EG$_4$, (g) EDOT-EG$_6$, (h) EDOT-EG$_2$OMe, (i) EDOT-EG$_4$OMe, and (j) EDOT-EG$_6$OMe.

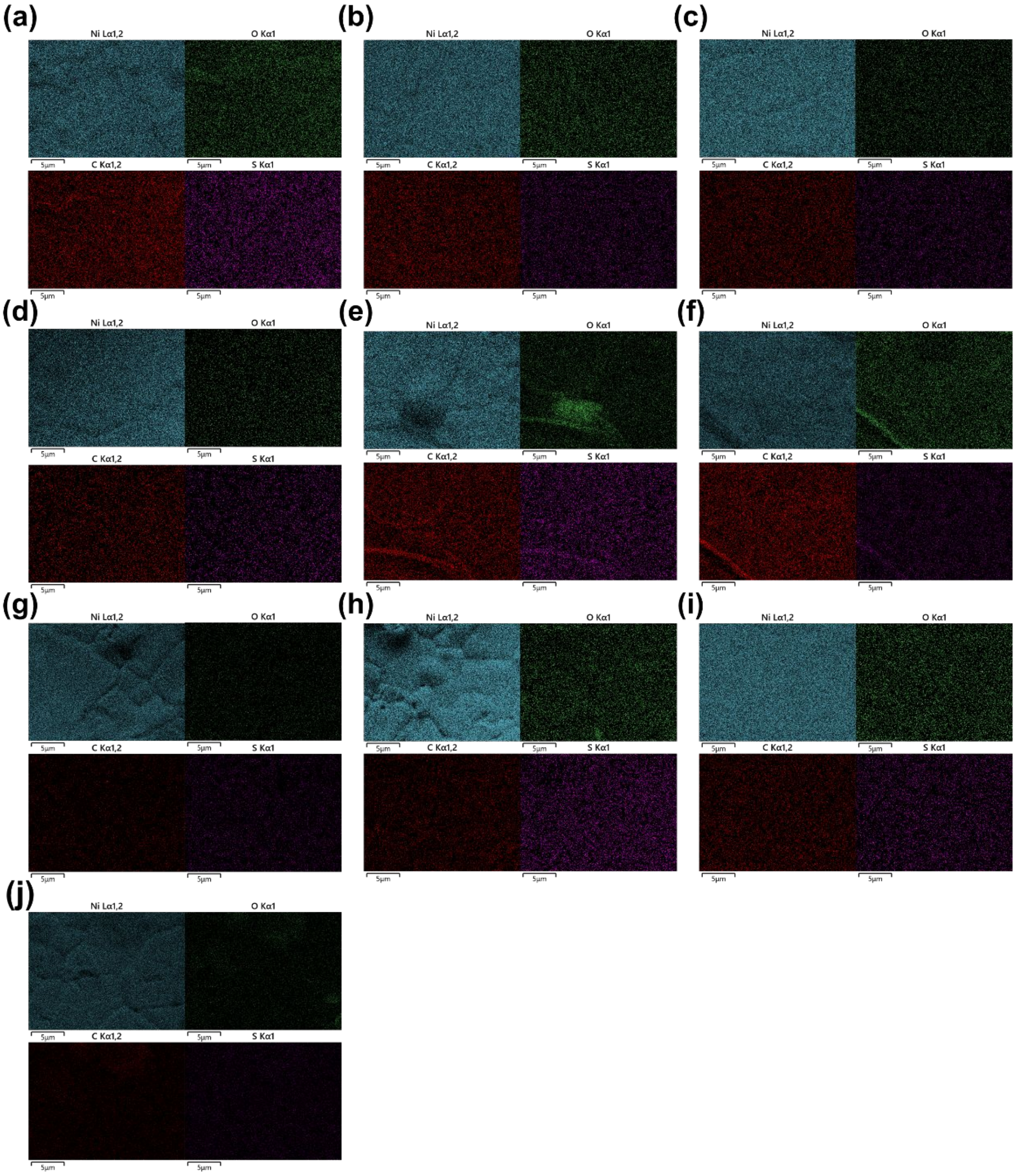

Figure S9. Energy dispersive X-ray spectroscopy (EDS) elemental mapping of (a) E-EG$_2$-E, (b) E-EG$_4$-E, (c) E-EG$_6$-E, (d) E-EG$_8$-E, (e) EDOT-EG$_2$, (f) EDOT-EG$_4$, (g) EDOT-EG$_6$, (h) EDOT-EG$_2$OMe, (i) EDOT-EG$_4$OMe, and (j) EDOT-EG$_6$OMe.

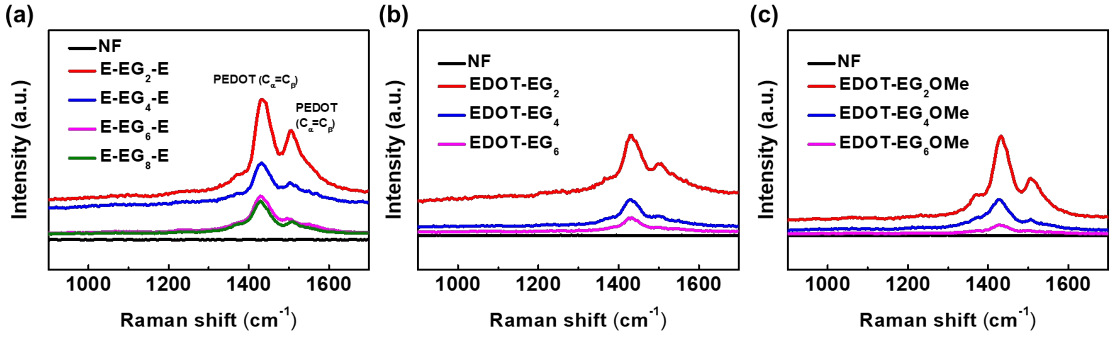

Figure S10. Raman spectra of (a) E-EG$_n$-E, (b) EDOT-EG$_n$, and (c) EDOT-EG$_n$OMe.

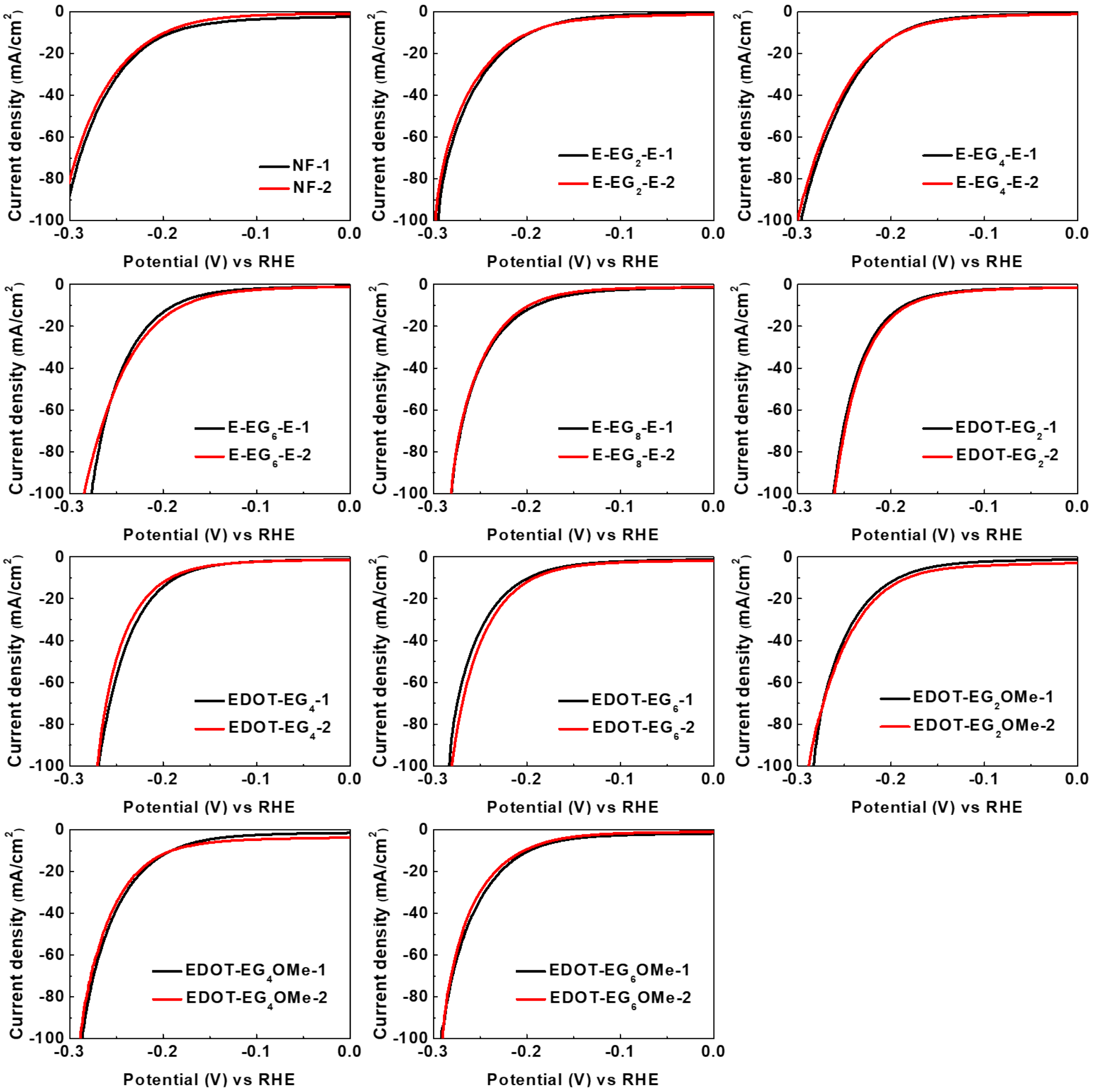

Figure S11. Repeated LSV curves of EG-functionalized EDOTs.

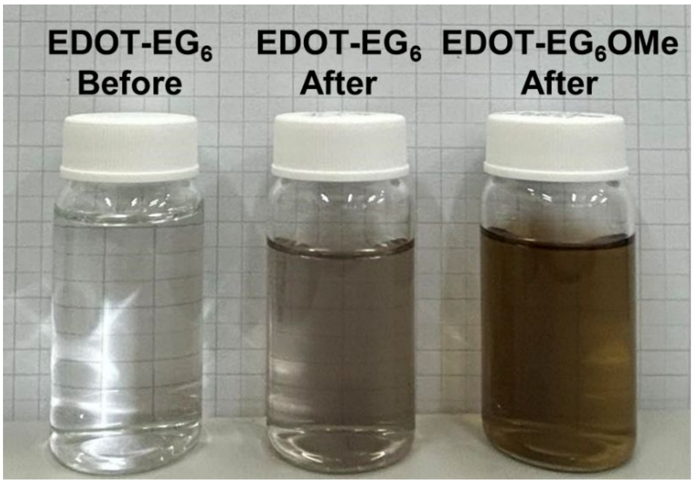

Figure S12. Solution containing EDOT-EG$_6$ before electropolymerization and solutions containing EDOT-EG$_6$, EDOT-EG$_6$OMe after electropolymerization.

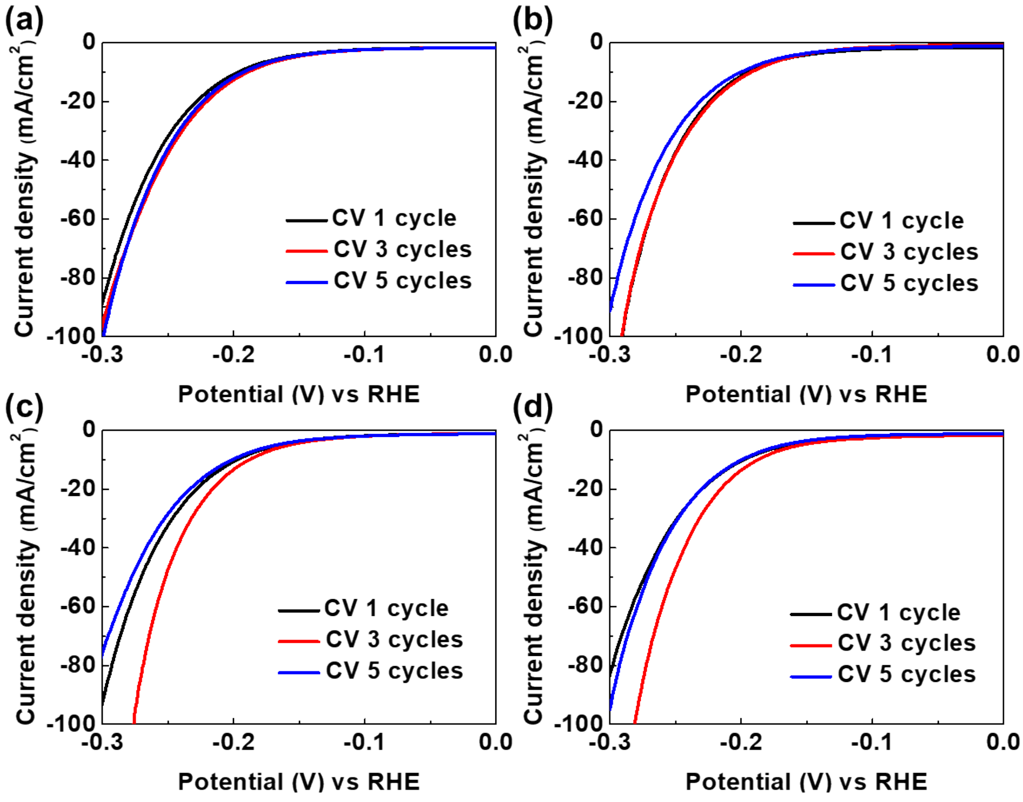

Figure S13. LSV results of (a) E-EG$_2$-E, (b) E-EG$_4$-E, (c) E-EG$_6$-E, and (d) E-EG$_8$-E with different cycles of potential scan during electropolymerization.

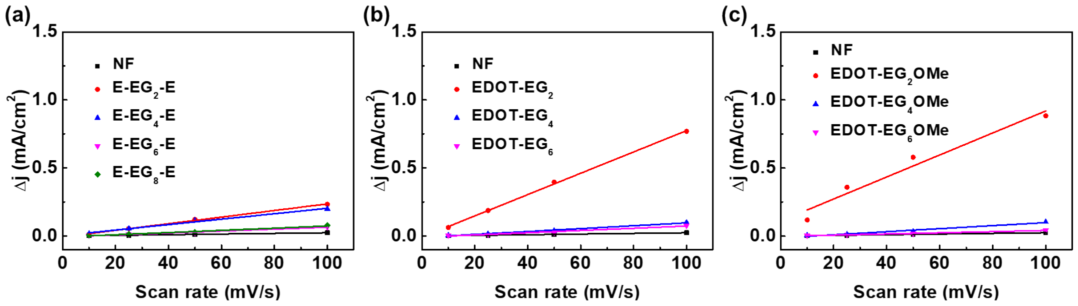

Figure S14. Current density vs scan rate plots of (a) E-EG$_n$-E, (b) EDOT-EG$_n$, and (c) EDOT-EG$_n$OMe.

Table S1. Calculated ECSA values.

Sample	ECSA (cm^2)
**NF**	6.1
**E- EG$_2$-E**	60.8
**E- EG$_4$-E**	50.0
**E- EG$_6$-E**	17.7
**E- EG$_8$-E**	20.1
**EDOT-EG$_2$**	196
**EDOT-EG$_4$**	26.5
**EDOT-EG$_6$**	20.4
**EDOT-EG$_2$OMe**	202
**EDOT-EG$_4$OMe**	28.0
**EDOT-EG$_6$OMe**	10.7

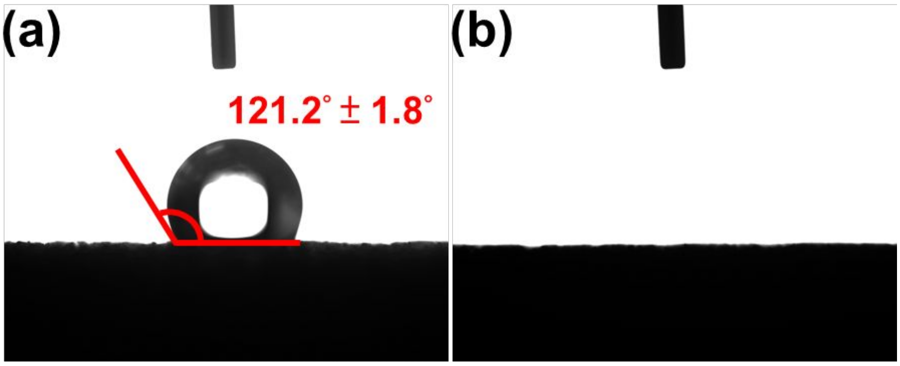

Figure S15. Water contact angles of (a) Blank NF, (b) poly(EDOT-OH) on NF.

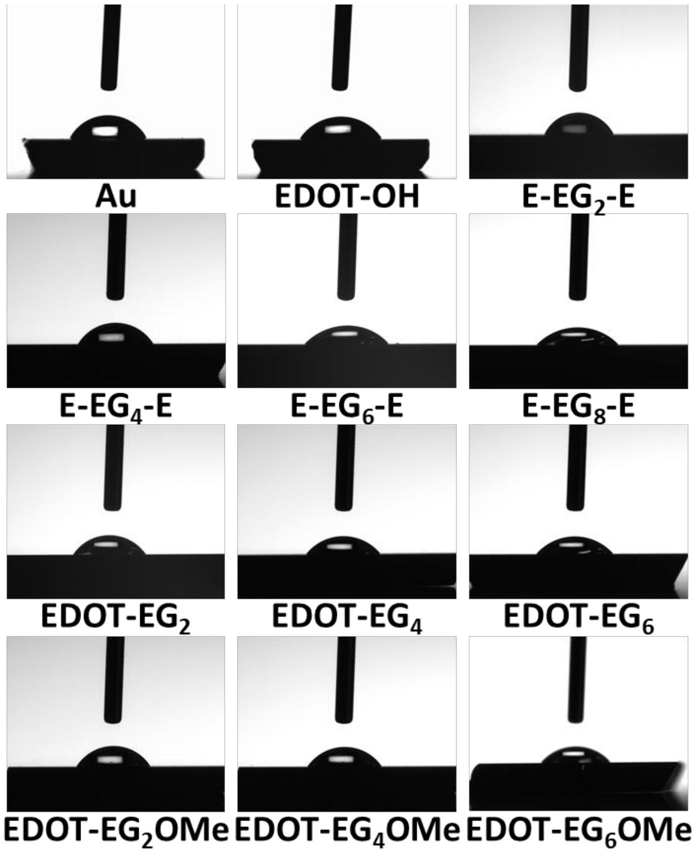

Figure S16. Optical images of water droplets on EG-functionalized EDOTs.

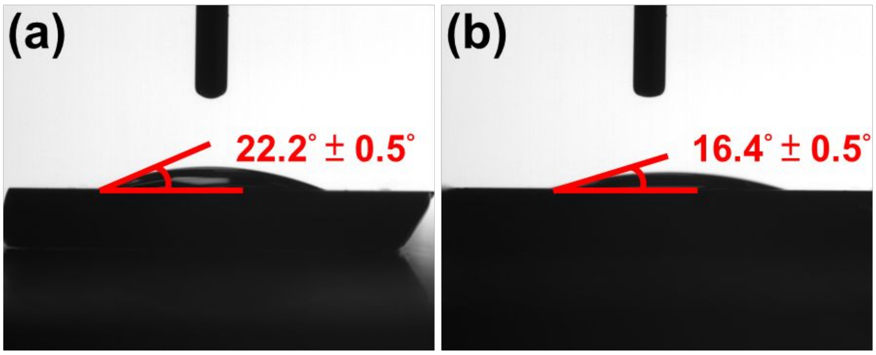

Figure S17. Water contact angles of (a) EDOT-S, (b) EDOT-PC on Au.

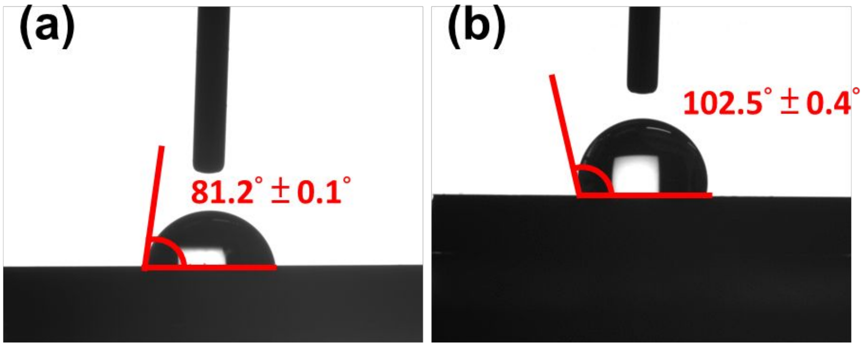

Figure S18. Water contact angles of (a) E-C_{12}-E, (b) EDOT-C_{12} on Au.

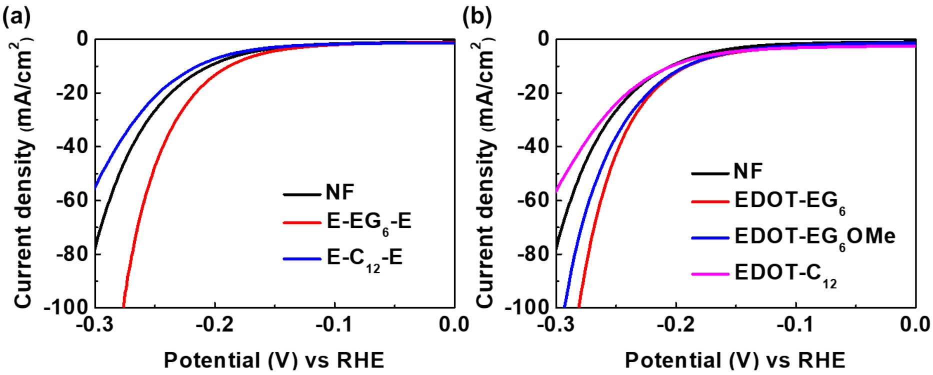

Figure S19. LSV curves of (a) E-C_{12}-E, (b) EDOT-C_{12}. LSV curves of blank NF, E-EG_6-E, EDOT-EG_6, and EDOT-EG_6OMe are presented for comparison.

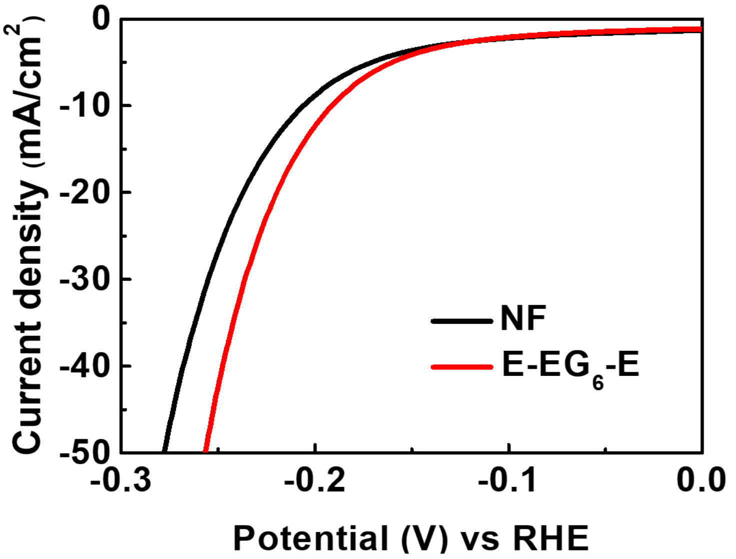

Figure S20. LSV curves of blank NF and E-EG$_6$-E with a GC counter electrode during measurements.

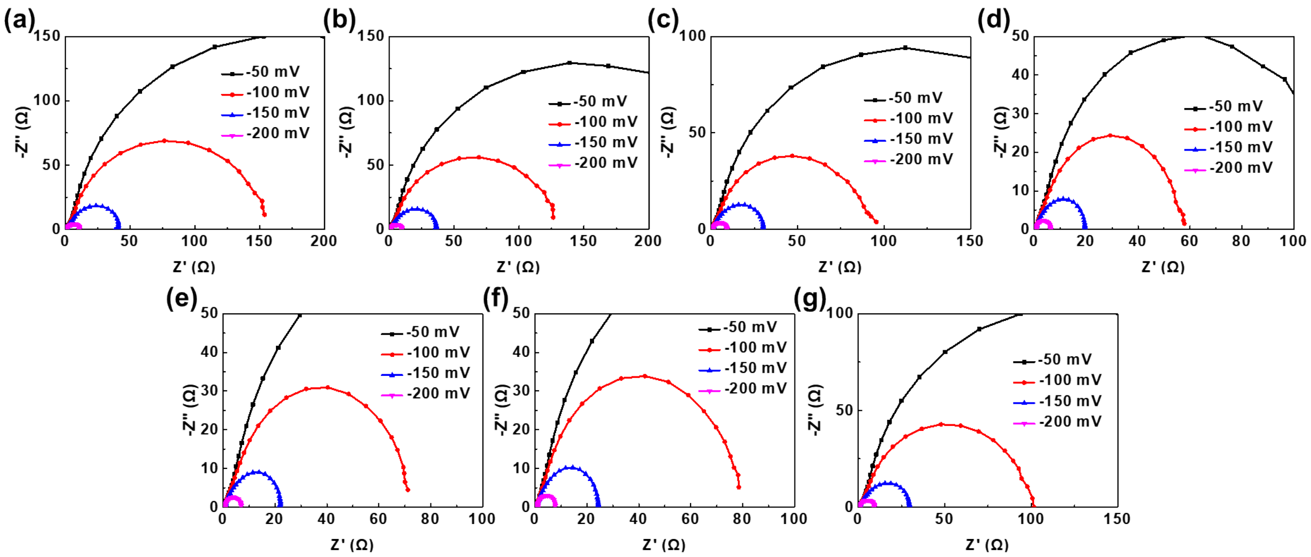

Figure S21. Nyquist plots of (a) Blank NF, (b) E-EG$_2$-E, (c) E-EG$_4$-E, (d) E-EG$_6$-E, (e) E-EG$_8$-E, (f) EDOT-EG$_6$, and (g) EDOT-EG$_6$OMe.

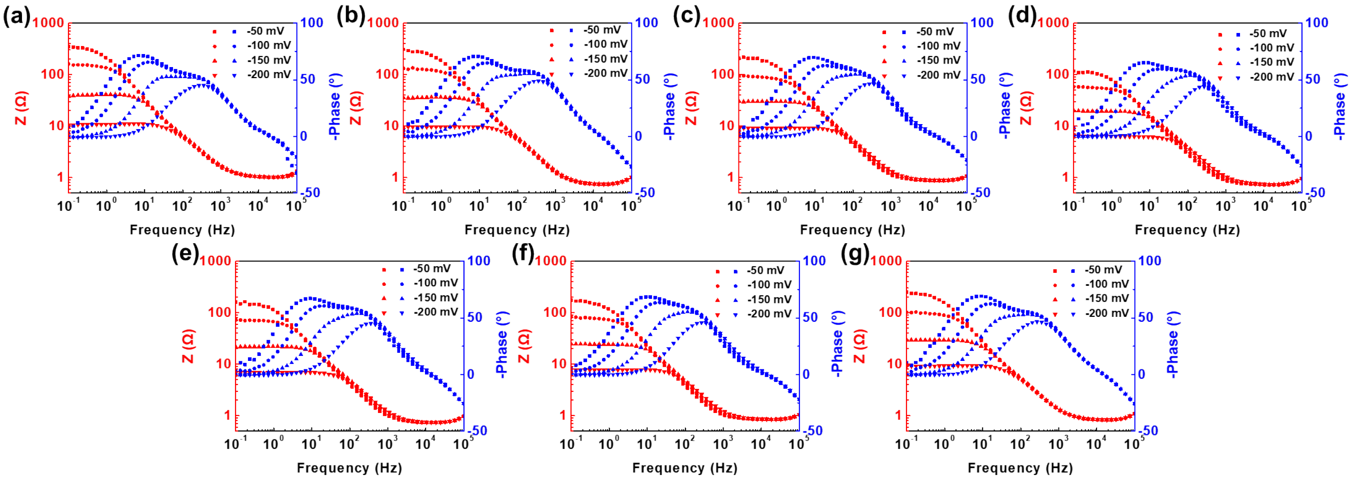

Figure S22. Bode plots of (a) Blank NF, (b) E-EG$_2$-E, (c) E-EG$_4$-E, (d) E-EG$_6$-E, (e) E-EG$_8$-E, (f) EDOT-EG$_6$, and (g) EDOT-EG$_6$OMe.

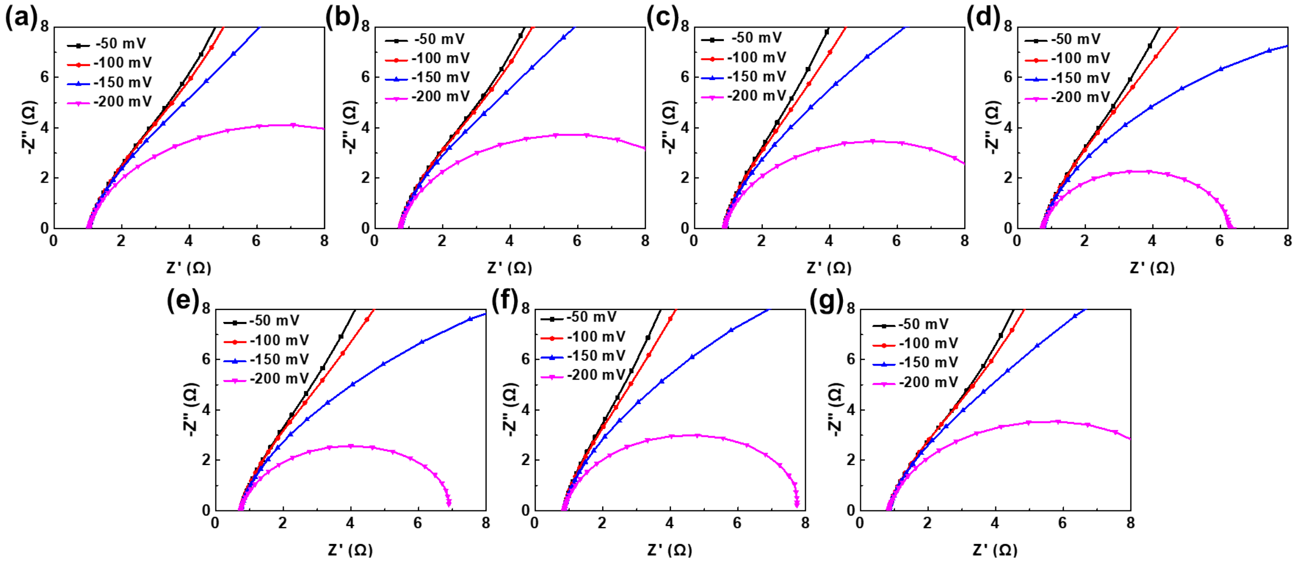

Figure S23. Nyquist plots of (a) Blank NF, (b) E-EG$_2$-E, (c) E-EG$_4$-E, (d) E-EG$_6$-E, (e) E-EG$_8$-E, (f) EDOT-EG$_6$, and (g) EDOT-EG$_6$OMe. The data are identical to Figure S17, with different scales.

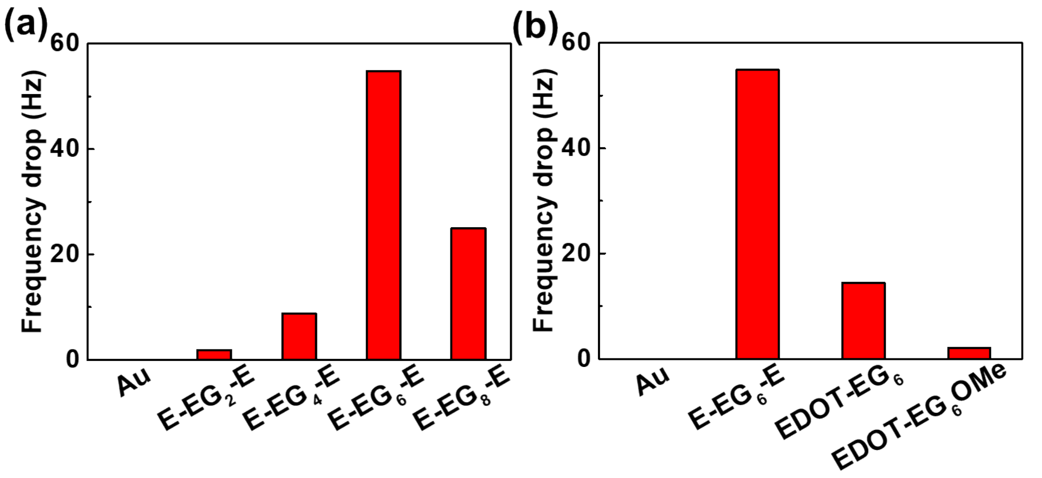

Figure S24. **Δf values at 40 min of (a) E-EG$_n$-E. (b) EG-functionalized EDOTs with 6EG groups.**

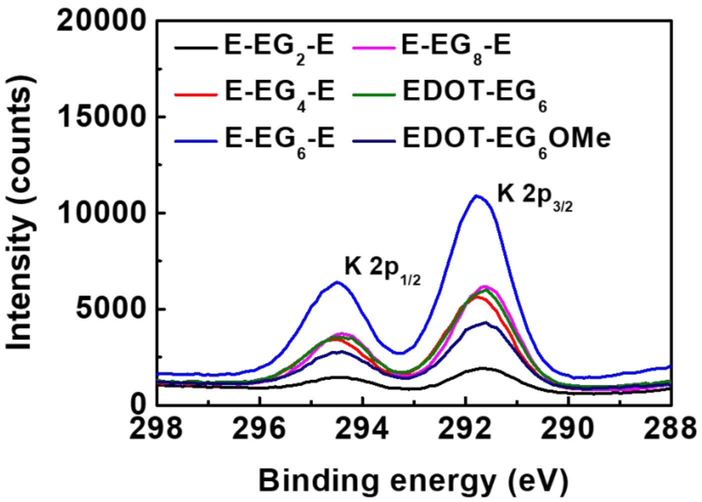

Figure S25. **K 2p XPS spectra of E-EG$_2$-E, E-EG$_4$-E, E-EG$_6$-E, E-EG$_8$-E, EDOT-EG$_6$, and EDOT-EG$_6$OMe.**

Table S2. Calculated area ratios from the K 2p XPS spectra. The area from the XPS spectra was divided by the area of E-EG$_6$-E.

Sample	Area ratio
E- EG$_2$-E	0.109
E- EG$_4$-E	0.486
E- EG$_6$-E	1
E- EG$_8$-E	0.558
EDOT-EG$_6$	0.533
EDOT-EG$_6$OMe	0.355

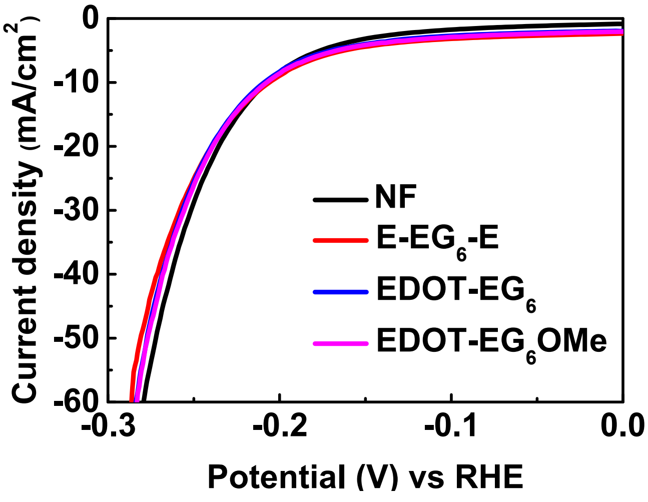

Figure S26. LSV curves of NF, E-EG$_6$-E, EDOT-EG$_6$, and EDOT-EG$_6$OMe in TMAH solution.
